# A theory of how active behavior stabilises neural activity: Neural gain modulation by closed-loop environmental feedback

**DOI:** 10.1371/journal.pcbi.1005926

**Published:** 2018-01-17

**Authors:** Christopher L. Buckley, Taro Toyoizumi

**Affiliations:** 1 Laboratory for Neural Computation and Adaptation, RIKEN Brain Science Institute, Saitama, Japan; 2 Department of Informatics and Engineering, University of Sussex, Falmer, United Kingdom; University College London, UNITED KINGDOM

## Abstract

During active behaviours like running, swimming, whisking or sniffing, motor actions shape sensory input and sensory percepts guide future motor commands. Ongoing cycles of sensory and motor processing constitute a closed-loop feedback system which is central to motor control and, it has been argued, for perceptual processes. This closed-loop feedback is mediated by brainwide neural circuits but how the presence of feedback signals impacts on the dynamics and function of neurons is not well understood. Here we present a simple theory suggesting that closed-loop feedback between the brain/body/environment can modulate neural gain and, consequently, change endogenous neural fluctuations and responses to sensory input. We support this theory with modeling and data analysis in two vertebrate systems. First, in a model of rodent whisking we show that negative feedback mediated by whisking vibrissa can suppress coherent neural fluctuations and neural responses to sensory input in the barrel cortex. We argue this suppression provides an appealing account of a *brain state transition* (a marked change in global brain activity) coincident with the onset of whisking in rodents. Moreover, this mechanism suggests a novel signal detection mechanism that selectively accentuates *active*, rather than *passive*, whisker touch signals. This mechanism is consistent with a predictive coding strategy that is sensitive to the consequences of motor actions rather than the difference between the predicted and actual sensory input. We further support the theory by re-analysing previously published two-photon data recorded in zebrafish larvae performing closed-loop optomotor behaviour in a virtual swim simulator. We show, as predicted by this theory, that the degree to which each cell contributes in linking sensory and motor signals well explains how much its neural fluctuations are suppressed by closed-loop optomotor behaviour. More generally we argue that our results demonstrate the dependence of neural fluctuations, across the brain, on closed-loop brain/body/environment interactions strongly supporting the idea that brain function cannot be fully understood through open-loop approaches alone.

## Author summary

Animals actively exploring or interacting with their surroundings must process a cyclical flow of information from the environment through sensory receptors, the central nervous system, the musculoskeletal system and back to the environment. This closed-loop sensorimotor system is essential for an animal's ability to adapt and survive in complex environments. Importantly, closed loop feedback signals also regulate brainwide neural circuits for behavior. Specifically, the activity of coherent populations of neurons inform motor behaviours and in turn are influenced by sensory feedback signals mediated by the environment. We develop a theory that suggests that this feedback can explain the marked changes in large-scale neural dynamics and sensory processing (together referred to as brain state) that coincide with the onset of active behaviours. This feedback may contribute to flexible context dependent neural computations in brain systems.

## Introduction

Neural response are strongly sensitive to behavioural state. The onset of movement such as running and whisking is coincident with prominent modulations in neural activity in sensory areas [1–3]. The rodent whisker system has become a key model system within which to investigate these changes [4–6]. The onset of active whisking in a previously quiet but attentive rodent is correlated with a marked reduction in endogenous synchronous neural activity of neurons in sensory areas; quantified as a reduction in low frequency fluctuations and a decrease in correlations between the membrane potentials of neurons in the barrel cortex [4]. Furthermore, membrane potential responses to experimentally induced perturbations of the whisker are also reduced by the presence of whisking [6]. These changes suggest that movement reduces neural gain [7,8] in the barrel cortex suppressing neural fluctuations and sensory response. Several internal pathways have been implicated in this gain regulation including various neuromodulatory pathways [9,10], intracortical feedback modulation by motor areas [11] or they could be directly triggered by changes in sensory input [12,13] via thalamo-cortical projections [14]. Despite this gain reduction, robust responses to sensory input occur during *active contact events* when the whisker collides with an object placed in the whisk field [5,6]. Thus, a whisking-induced gain reduction cannot by itself account for the difference in sensory responses to whisker perturbations and active contact events without appeal to additional mechanisms [15]. The reafference principle (RP) [16] also does not straightforwardly explain these differences. The RP explains the amplitude of sensory response by a mismatch between the actual sensory input and its prediction, where the prediction is based on an *efference copy* (an internal copy of motor command). But the RP does not explain why sensory responses to whisker perturbations, which are always unpredicted, are suppressed during movement.

Active behaviours are defined by closed-loop feedback interactions between brain/body/environment which are central to motor control and, it has been argued, pivotal to account of perceptual processes [17–19]. During active whisking *reafferent* sensory input (sensory input resulting from one's own actions) conveys information about proprioceptive sensory feedback of whisking and which informs the subsequent motor control of the vibrissae [20,21]. Repeated cycles of reafferent sensory input followed by motor output constitute a closed-loop feedback interaction between cells in the barrel cortex and the vibrissae [22]. In this work, we show that in this system closed-loop feedback mediated by whisking vibrissae can: 1. Suppress synchronous endogenous neural fluctuations and passive sensory responses, 2. Account for large response to active touch events because of a transient interruption of this feedback. The results provide a nuanced view of predictive coding where neurons represent predictions errors about consequences of motor actions rather than the difference between the predicted and actual sensory input. More generally these results strongly support the centrality of closed-loop interaction in perceptual apparatus [17] by suggesting a specific role they play in event detection.

To support a key prediction of this theory we examine how closed-loop interactions in a motor control behaviour impact on neuronal fluctuations. Specifically, we re-analysed data from a second system, a larval zebrafish behaving in a virtual reality where fictive water flow is simulated by a grating (striped image) drifting across the fish retina [23]. In this set up zebrafish larvae are immobilised with a neuromuscular blocker. The fish's attempted movements relative to the grating are monitored through motor neuron activity and translated into appropriate modulation of the velocity of the grating [23]. With data from this setup we show that the presence of closed-loop interactions between neurons and fictive swim speed causes the suppression of synchronous neural fluctuations across the fish brain in a manner analogous with the rodent whisker system. Further we show that the amount of this suppression for each neuron is correlated with the strength of its involvement in the optomotor signaling. Together, these results suggest that understanding changes in neural activity across the brain caused by the onset of movement requires the study of closed-loop brain/body/environment interactions beyond open-loop sensory paradigms. Thus we strongly support the argument that a full understanding of phenomenology of neural circuits during active behaviors requires moving away from the idealisation of the brain as an input/output information processor toward its role as a dynamic control system regulating behaviour [19].

## Results

### Theory

**Fig 1:**
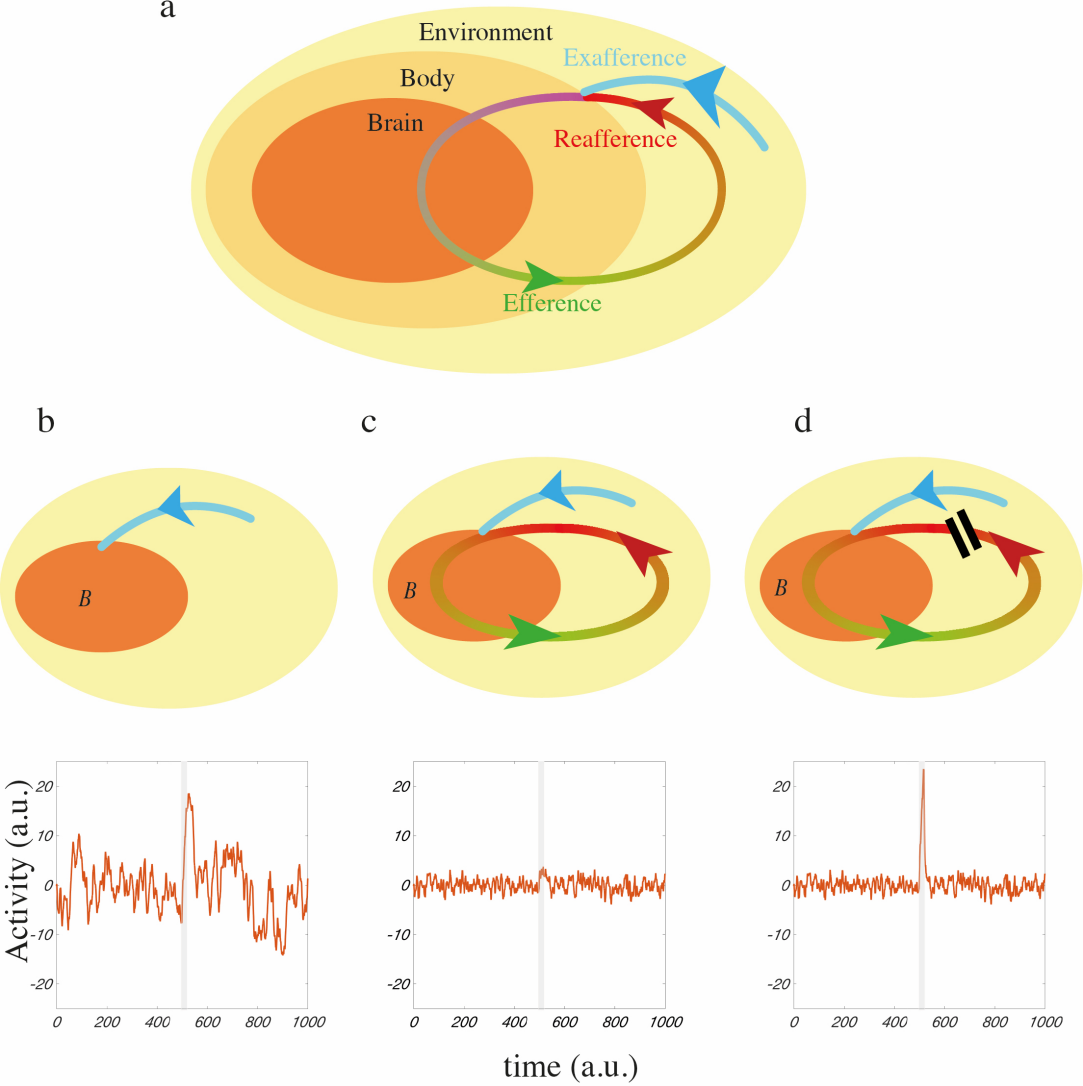
A simple model of the brain-environment interaction. **a**, A schematic description of brain-body-environment interactions during closed-loop behavior. The brain receives two types of sensory input: exafferent input (blue) that originates from the environment and reafferent input (red), which, while mediated by the environment, results from the consequences of an animal’s own actions. (**b-d**) Schematic diagrams of the interactions between the brain (B) and the body/environment with example neural activity traces of the simple model described in the text with *τ* = 1.05 and *w* = −0.5. Each model is Euler integrated (*dt* = 0.01) and is driven by normally distributed noise with zero mean and unit variance (the noise is slightly smoothed for presentation purposes). A perturbation (*I* = 2) is applied at t = 500 for 20 time units (grey bar). **b**, The brain receives no reafferent input (an open-loop condition) and exhibits collective activity that spontaneously fluctuates. The magnitude of the fluctuations and the responses to perturbation are large. **c**, Reafferent input mediates closed-loop sensory feedback to the brain (a closed-loop condition). If this feedback is negative, the gain of the brain is reduced and both fluctuations and responses to perturbation are suppressed. **d**, A perturbation in the closed-loop condition are combined with a brief interruption (20 time units) of the closed-loop feedback. Responses to perturbation are accentuated during the interruption but background fluctuations are suppressed before and after the contact.

In moving animals, the brain receives sensory input that originates in the external environment, or *exafferent* sensory input *(*Fig 1A, blue arc). In addition, efferent motor commands *(*Fig 1A, green arc) drive the body and environment and induce *reafferent* (self-generated) sensory input *(*Fig 1A, red arc) [16,24]. To develop an intuition of how closed-loop feedback, mediated by reafferent input, could impact on neural activity we introduce two model conditions. First, we assume that when an animal is not moving the brain receives only exafferent input, we describe this as an *open-loop* condition (Fig 1B, top). Second, when the animal begins to move the brain interacts with the environment coupling motor action and reafferent sensory input, we refer to this as a closed-loop condition (Fig 1C top). Note: it is likely that some reafferent input is always present but our focus here is on the effect that the onset of a previously absent reafferent sensory pathway could have on neural activity. We examine these two conditions in a simple idealized model, see [17] for a similar idealisation, where brain variable *B* (which describes collective neural activity, e.g., membrane potential activity) receive input from, or interacts with, the body and environment. In the open-loop condition the collective neural activity, *B*_*o*_(*t*), is assumed to be described in term of a first-order linear differential equation,

$$\frac{dB_{o}(t)}{dt}=\frac{-B_{o}(t)}{\tau }+{I(t)+\xi }_{o}(t),$$ (1)

where *ξ*_*o*_ is white noise of instantaneous variance *σ*^2^ generated inside the brain, *t* is time, *τ* is the time constant of the system and *I*(*t*) is exafferent input. Essentially, in the absence of input, we represent collective neural activity as a simple leaky integrator system with leak timescale *τ* driven by endogenous noise (see Fig 1B, bottom, for traces). Of interest here is the magnitude of fluctuations which can be calculated as the autocorrelation peak (instantaneous variance) of variable *B*_*o*_ which is *Peak*_*o*_ = *σ*^2^ *τ*/2, and the gain of the response to sensory input (calculated as the ratio between a static input and an equilibrium response), which is simply *Gain*_*o*_ = *τ*. Thus in this simple system both the gain and the fluctuations are determined by the timescale of the endogenous dynamics. However, during the closed-loop condition we write the dynamics of the brain variable,

$$\frac{{dB}_{c}(t)}{dt}=\frac{-B_{c}(t)}{\tau }+wB_{c}(t)+I(t)+\xi_{c}(t),$$ (2)

where we have idealised reafferent input as a simple self-feedback signal with strength *w*, i.e., we have assumed this feedback is linear and instantaneous (we will relax this assumption later). In this condition, the continuous cycles of reafferent input constitute a closed-loop feedback signal to the brain. The presence of this feedback changes the effective time constant to *τ*_*eff*_ = *τ*/(1−*wτ*). The magnitude of the fluctuations is now characterized by autocorrelation peak *Peak*_*c*_ = *Peak*_*o*_/(1−*wτ*) and the effective gain of the system is *Gain*_*c*_ = *Gain*_*o*_/(1−*wτ*). In particular, if this feedback is negative (*w* < 0), it will suppress both fluctuations and the gain of sensory responses, see Fig 1B and 1C (bottom panels). This very simple model suggests that, in principle, closed-loop feedback mediated by the body/environment could have a direct impact on neural activity. One way to accentuate sensory responses is described in Fig 1D. Here the brain initially has low closed-loop gain (*Gain*_*c*_ = *τ*/(1−*wτ*)) and thus exhibits suppressed fluctuations. However, if during a sensory event (Fig 1D, grey bar) closed-loop feedback is interrupted, e.g., if whisking is interrupted by contact with an object (see below), then brain will have temporarily high open-loop gain (*Gain*_*o*_ = *τ*). Thus the combination of a large sensory response and suppressed background fluctuations prior to sensory event can accentuate signal-to-noise ratios. In the following, we explain how these three conditions can be realized in the rodent whisker system.

### The rodent whisker system

#### The role of closed-loop feedback in a brain state transition

Does the presence of closed-loop sensory feedback explain the changes in neural activity caused by the onset of whisking in the rodent barrel cortex? We examined neural membrane potential recordings made in head-fixed rodents with all but a single vibrissae removed [4,6], In these experiments rodents transitioned between two behaviours: a quiet attentive behaviour (i.e. awake and not sleeping, but with stationary vibrissae) or spontaneous bouts of whisking [4,6]. In the absence of whisking the membrane potential of neurons in the barrel cortex exhibit noisy fluctuations with a strong 1Hz component and correlations between neighbouring neurons are relatively high [4]. The onset of active whisking suppresses these coherent neural fluctuations. In addition the sensory responses of neurons to passive whisker deflections are also suppressed by whisking [4,6]. However, neurons robustly respond to active touch events when the whisker collides with, and briefly comes to rest on, an object placed in the whisk field [4,6]. Furthermore, the coherent neural fluctuations reappear if the whisker repeatedly collides with an object [6].

While the presence of closed-loop sensory feedback is a major difference between the quiet attentive and active whisking states, it has been shown that coherent fluctuation of membrane potential are suppressed by whisking behavior even when the infra-orbital nerve (ION) is cut, removing sensory input from whiskers [5]. At first glance, the effect of this lesion seems to rule out a role for closed-loop sensory feedback. Accordingly, we made a closer examination of the role sensory input plays in this suppression by further analysing the data from this ION cut experiment, data supplied by the authors of [5]. We found that the latency between the onset of whisking and the suppression of membrane potential fluctuations was longer under the ION cut condition, as compared to the ION intact condition (see S1 Appendix). Thus, while there are likely many internal mechanisms underlying brain state transitions such as thalamo-cortical input [25] or corollary discharge [26] this analysis suggests a role for sensory input under physiological conditions.

#### A model of cortical-vibrissae interactions

**Fig 2:**
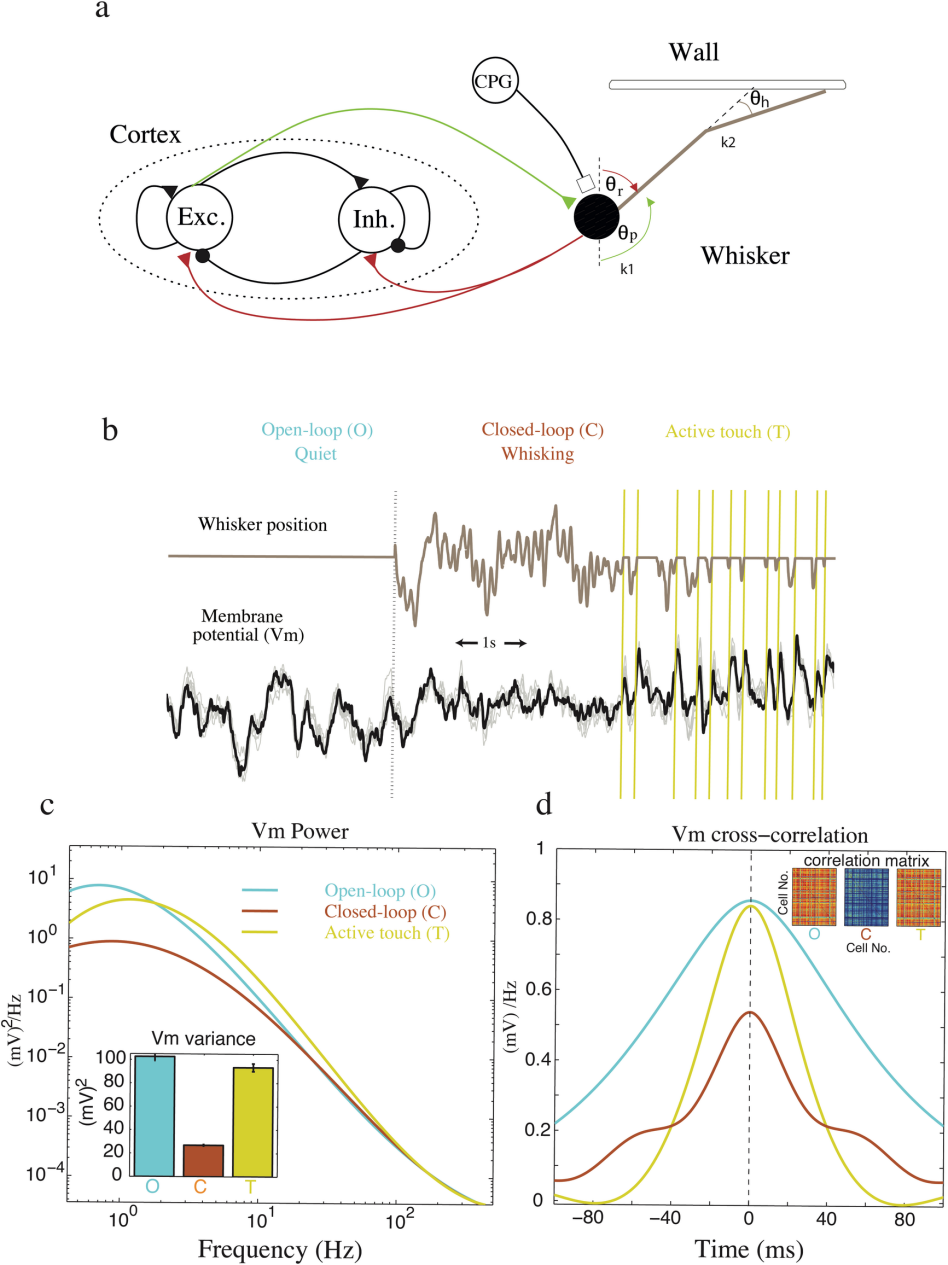
Coherent fluctuations are suppressed by the presence of whisking in a simple model of the barrel cortex. **a**, A schematic of a simplified whisking model. 100 excitatory (Exc) and 100 inhibitory (Inh.) neurons receive sensory feedback via a single whisker driven by a central pattern generator (CPG) and the excitatory neurons. Triangle and circles represent excitatory and inhibitory synapse respectively. Onset of whisking occurs when the CPG is switched on. Sensory feedback is negative overall because the neurons that elicit whisker protraction are assumed to be driven by whisker retraction. The whisker comprise of two sections with a bending angle, *θ*_*h*_, and protraction angle *θ*_*p*_. The base and tip sections are constrained by two springs with spring constants *k*1 and *k*2 respectively. Whisking is implemented by driving the equilibrium position of the base spring. The center spring is in equilibrium at zero angular displacement and tends to align the whisker sections. The whisker is unit length and massless but constrained by a solid wall placed within the whisker’s reach. **b**, Membrane potential of cortical neurons (light lines for individual neurons and dark line for population average) and whisker position in a quiet attentive (open-loop condition: O) and whisking (closed-loop condition: C) rodents. Periods of active touch (T) are also shown. Large and synchronous fluctuations of membrane potential were suppressed during whisking. Active touch elicited reliable responses in these neurons. The vertical dotted line marks the onset of whisking and the vertical solid lines mark onset of individual touch event. **c**, The power spectrum (inset for variance) of membrane potential are averaged over cortical neurons and shown for each condition. **d**, Similarly cross-correlation (inset for correlation matrix of randomly sampled neurons—color warmth indicates the degree of correlation) of membrane potential are averaged over cortical neurons and shown for each condition. Coherent low frequency fluctuations and inter-neural correlation are suppressed during C relative to O but are recovered during T.

To test whether closed-loop feedback could plausibly explain the changes in brain activity caused by whisking we constructed a simplified neural network model of cortical-vibrissae interactions (see Materials and Methods). Our model comprises both an excitatory and inhibitory cortical population dynamically interacting with a single vibrissa (Fig 2A). Slow coherent fluctuations of membrane potential at around 1Hz arise in this model from an interplay between the buildup of excitatory cortical activity through recurrent activity and their eventual suppression by adaptation after ca. 1 s in each cell. We model a simple flexible vibrissa as two hinged stiff mass-less sections of unit length with relative angle *θ*_*h*_ (bending angle) connected at the base with protraction angle *θ*_*p*_ to the body. The sections are constrained by simple torsion springs with spring constant *k*_1_ and *k*_2_ respectively, see Fig 2A. The center spring has an equilibrium value of zero angular displacement and thus tends to align both sections (see Materials and Methods) and whisker movement is driven by setting equilibrium position of the base spring. Fast whisking behavior is manually turned on or off by a central pattern generator (CPG) that periodically drives the whisker (~10 Hz) when CPG is on. Our theory (see Fig 1) suggests that negative sensory feedback could suppress coherent neural fluctuations. To model this, we assumed that cortical excitatory neurons additionally drive protraction of the vibrissae, and, in turn, both excitatory and inhibitory populations receive the sensory feedback of the whisker retraction angle *θ*_*r*_ = 180° − *θ*_*p*._ To model contact events a horizontal frictionless solid wall is placed above the whisker and, as the whisker collides with the wall, the whisker tip stops. In addition, we model a contact-detection signal capturing the stereotypical response of pressure sensitive cells in the trigeminal ganglion [27]. Specifically, we deliver a brief square wave pulses (ca. 25 ms) triggered by each whisker contact event as an additional sensory input (see Materials and Methods). See the Discussion and S2 Appendix, for further discussion of the biological plausibility of these model assumptions.

During the open-loop condition (i.e., the quiet attentive condition) the cortex exhibits significant synchronous low frequency membrane potential fluctuations which are suppressed in the closed-loop condition (i.e., the freely whisking condition). Like the simple mode in Fig 1, negative sensory feedback reduced the gain of the cortical system and replaced prominent (ca. 1 Hz) synchronous fluctuations of the membrane potential with fast (ca. 10 Hz), but weak, fluctuations locked into the whisking cycle, see Fig 2C. Furthermore, the average inter-neural correlation of membrane potential for pairs of neurons was also suppressed (Fig 2D) indicating that coherent fluctuations across the network were suppressed.

**Fig 3:**
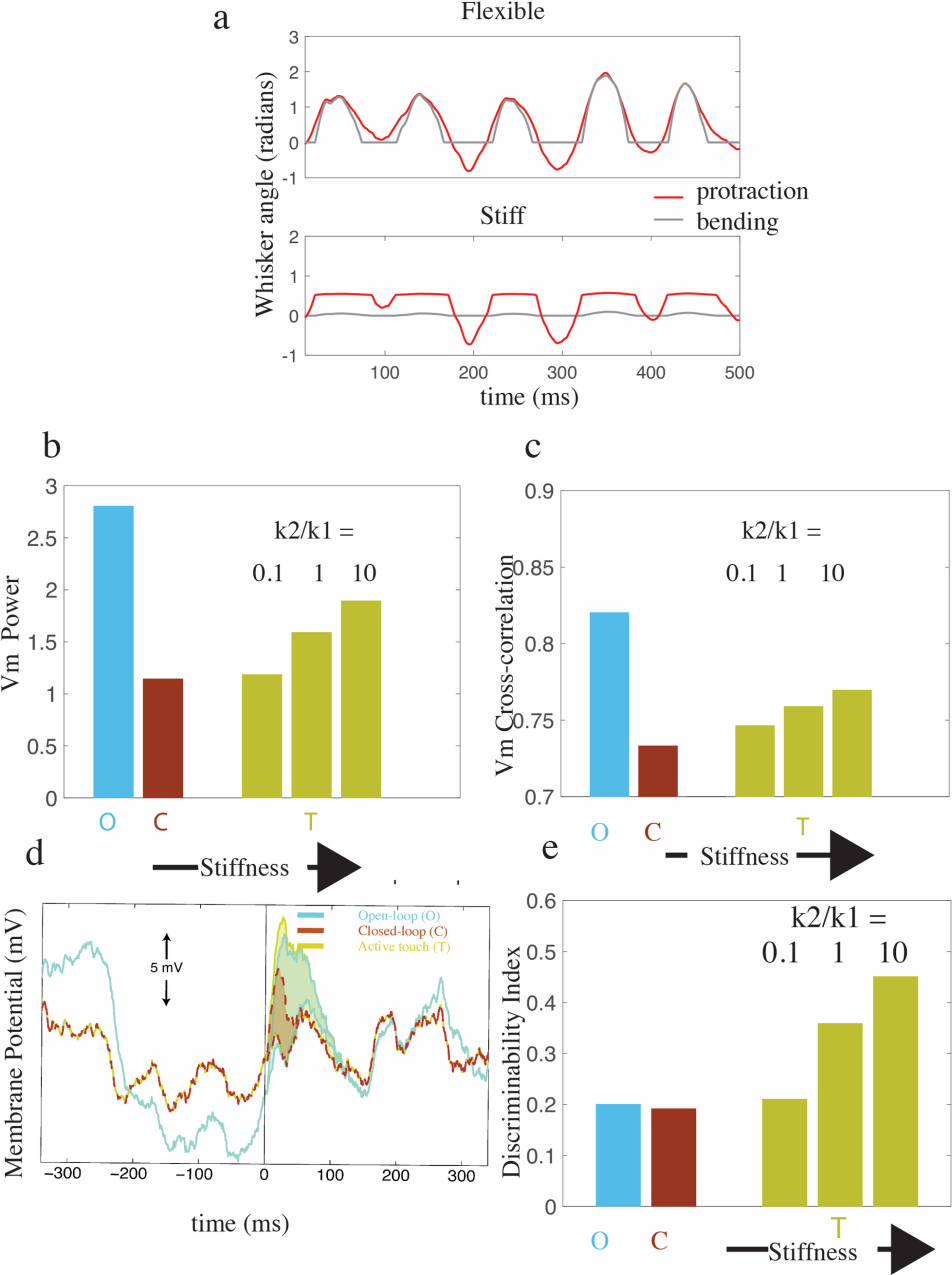
Negative closed-loop sensory feedback can account for the suppression of coherent fluctuations and sensory responses. **a**, The relationship between the bending (*θ*_*h*_: gray) and protraction angle (*θ*_*p*_: red) in a flexible (top, *k*2/*k*1 = .1) and stiff (bottom, *k*2/*k*1 = 10) whisker. The deflection of the protraction angle is smaller during contact if the whisker is stiff. **b** and **c**, The dependence of low frequency power and intra-neural correlation on behavioural condition (O and C) and whisker stiffness, respectively. Data averaged over 100 trials for each condition. In all cases standard error in the mean was much smaller than 1% of the mean. **d,** A comparison of the Membrane potential response to exafferent input during O (blue), C (red) and an active touch event (yellow). Events begin at time 0. Active touch has the same baseline as C but a similar response magnitude to O. **e,** The dependence of the discriminability index (see Materials and Methods) on behavioural condition (O and C) and increasing whisker stiffness. Discrimination performance was improved with increasing whisker stiffness, reflecting the degree to which closed-loop sensory feedback was interrupted during touch events.

We then examined the effect of whisker contact events on cortical dynamics. If we assume the whisker is perfectly rigid and unbending then the whisker stops when it comes into contact with the object interrupting sensory feedback as suggested in Fig 1D, see Fig 2A (grey line) where touch events are marked by yellow lines. During periods of contact events the frequency power and cross-correlation of membrane potential fluctuations were partially recovered, see Fig 2C and 2D (yellow lines), in agreement with experimental results [4,6]. We hypothesized that if the whisker is very flexible, then the protraction angle would change continuously, despite contact of the tip, fully preserving sensory feedback of whisker movement. Note: to distinguish the effect of sensory feedback from touch-evoked signal, we applied the same square wave pulse as a contact-detection signal regardless of whisker stiffness. We found that the recovery of 1–5 Hz power was stronger for a stiff whisker indicating that the interruption of the closed-loop sensory feedback was important for this change, see Fig 3B and 3C.

#### A model of the impact of closed-loop feedback on sensory processing

The same model also accounted for behavior-dependent changes in sensory processing [6] without assuming additional mechanisms [15,28]. Specifically, in agreement with experimental results, we found that exafferent perturbation evoked large sensory responses in the quiet condition but markedly smaller responses during the whisking condition (Fig 3D, [6]). Again, this was because negative closed-loop sensory feedback decreased the gain of the cortical circuit. Furthermore, in agreement with experimental data, we also found that cortical neurons exhibited more reliable responses to active touch events (i.e., events defined by a contact-detection signal in addition to a clipping of the whisker angle) than the same contact-detection signal during free whisking (Figs 2B and 3D). To quantify this effect in our whisker model, we computed a discriminability index (see Materials Methods) that characterize the signal-to-noise ratio of the response of the cortex to different sensory events. Effectively, this index measures the separation between the distributions of membrane potentials in the presence or absence of sensory events and is high when an event is very discriminable from background. The value of the index was similar for exafferent perturbations in the closed-loop and open-loop conditions, Fig 3E (blue and red bars), i.e., although the deflection-evoked response (signal) was greater in the open-loop condition, so were background fluctuations of membrane potential (noise). In contrast, the discriminability index was greater for contact events, Fig 3E, yellow bars. We hypothesized this was because negative sensory feedback, that suppressed neural fluctuations during whisking, was transiently removed during active touch events allowing endogenous recurrent excitation to amplify the cortical response to the contact-detection signal (Fig 3D). Thus, active touch events combined large sensory evoked responses (signal) and low background fluctuations (noise), which is beneficial for information coding. To confirm that this increase in discriminability was because of the transient interruption of the closed-loop feedback, we simulated vibrissae of different stiffness. The result shows that active touch event are more discriminable when the whisker is stiff, suggesting the benefit of a well-timed interruption of the closed-loop feedback for amplifying cortical response, see Fig 3D (c.f. Fig 1D). Hence, this model suggests that cortical neurons are selectively sensitive to the interruption of the animal’s own active sensing.

### Zebrafish virtual reality

#### A closed-loop versus replay condition

**Fig 4:**
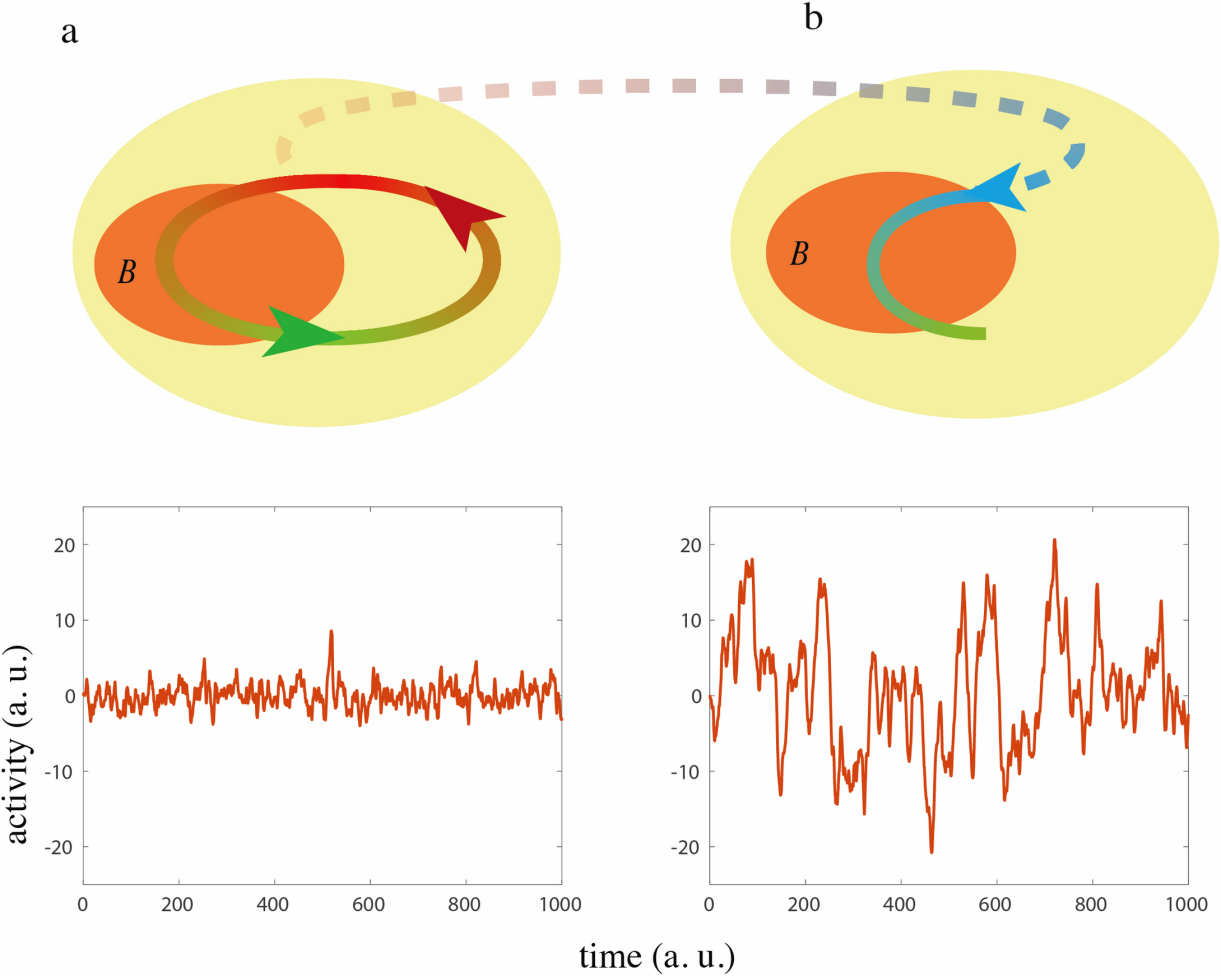
A theoretical prediction for the replay condition. Schematic diagrams and example traces of the simple model described in the text with *τ* = 1.05 and *w* = −0.5, same labelling conventions as Fig 1. Each model is Euler integrated (*dt* = 0.01) and is driven by normally distributed noise with zero mean and unit variance (the noise is slightly smoothed for presentation purposes). **a,** Reafferent input mediates closed-loop sensory feedback to the brain (a closed-loop condition: top panel) suppressing fluctuations in brain activity (bottom panel). **b,** the brain receives a replay of the reafferent input received in the closed-loop condition as exafferent input (top panel). Any differences from the closed-loop condition are caused by the absence of feedback because the sensory input is identical to that in the closed-loop condition. Note the noise is unique in each condition but sampled from the same distribution. In the replay condition fluctuations are much larger than in the closed-loop condition because of the absence of negative feedback.

Our theory makes the strong prediction that brain dynamics are sensitive to closed-loop feedback rather than sensory input per se. To illustrate this prediction we returned to the simple conceptual model presented in Fig 1 and introduce a *replay condition*. In this condition sensory input to the brain in a closed-loop condition is first recorded. This recording is then replayed in open-loop (i.e. as exafferent input) to an identical brain albeit with a different instantiation of internal noise, see Fig 4. Under this condition the dynamics of the brain, *B*_*r*_, can be written as:

$$\frac{dB_{r}(t)}{dt}=\frac{-B_{r}(t)}{\tau }+wB_{c}(t)+\xi_{r}(t),$$ (3)

where *ξ*_*r*_ is again white noise of instantaneous variance, *σ*^2^, that has the same statistics as the closed-loop condition. Here, the brain receives the same sensory input as in the closed-loop condition (Eq 2), i.e. *wB*_*c*_(*t*), but this time as exafferent input rather than reafferent input i.e. sensory input depends on *B*_*c*_ not *B*_*r*_ and thus is not real-time feedback. The magnitude of the fluctuations in this condition, again calculated as the autocorrelation peak of the brain variable, is *Peak*_*r*_ = *Peak*_*c*_ + *Peak*_*o*_ * 2 *wτ*/(*wτ* −2). Thus, this simple model predicts that even though the brain receives exactly the same total sensory input in the replay and closed-loop conditions the amplitudes of fluctuations will not be the same. In particular, if this feedback is negative, we obtain *Peak*_*c*_ < *Peak*_*o*_ < *Peak*_*r*_, see Fig 1B and 1C and Fig 4A and 4B.

#### The effect of closed-loop feedback on brain-wide dynamics in a behaving zebrafish

**Fig 5:**
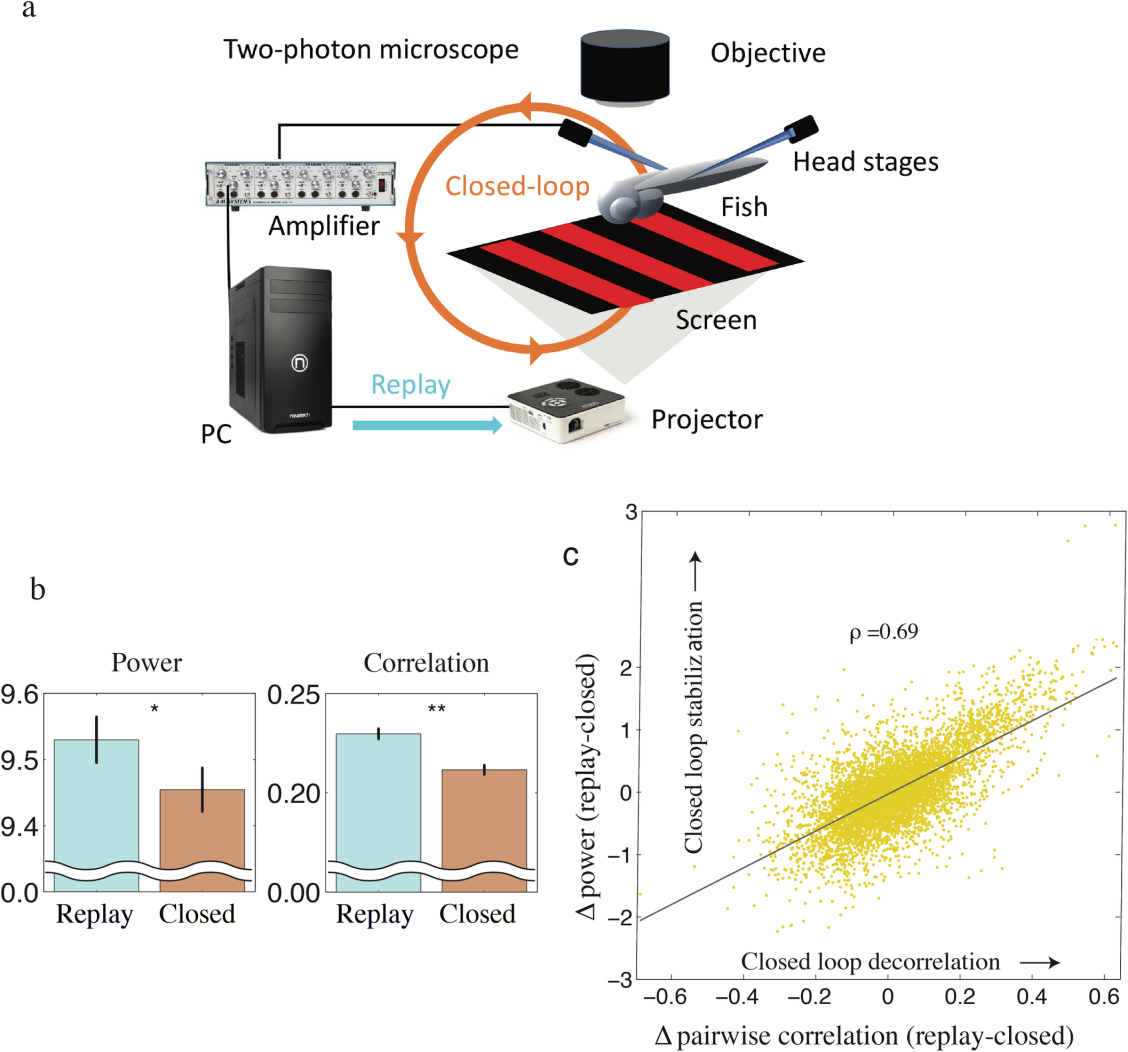
Synchronous neural fluctuation are smaller in the presence of closed-loop sensory feedback. **a** The experimental setup as described in [23]. In the closed-loop condition the grating stimulus is constructed based on motor nerve recording. In the replay condition the grating stimuli is a replay of stimulus seen in the closed-loop condition. **b**, Population averages of logarithmic low frequency power (mean over interval of [0.01 0.15] Hz) (left) and pairwise intra-neural correlations (right) were both suppressed under the closed-loop condition relative to the replay condition. **c,** These changes in pairwise correlations and the geometric mean of log low frequency power (replay–closed) were highly correlated in the recorded neurons.

To test this prediction we turned to a second experimental system, where we reanalyzed two-photon calcium imaging data recorded from larval zebrafish behaving in a virtual flow simulator, see introduction and [23] for full details. In this setup fish are immobilized with a neuromuscular blocker and fictive water flow is simulated by a grating (striped image) drifting across the fish retina (Fig 5A). Fictive swim bouts are then simulated by modulating grating speed based on the power of motor neuron activity (defined as swim power) recorded electrophysiologically from motor nerve along the spine of the fish [23]. Under natural conditions fish avoid being swept downstream by executing swim bouts (discrete bursts of swimming activity) in the direction opposite to water flow. Under VR conditions, oncoming water flow is simulated by allowing a grating to drift backward, in a tail-to-head direction, across the fish retina. Fish compensate for this drift with a specific optomotor behaviour in which fictive forward swim bouts temporally decelerate the grating maintaining their fictive horizontal position over time [23]. Despite neuromuscular blockade motoneuron firing is relatively normal under these conditions and fictive behaviours compare favourably with natural conditions [23,29]. During this behavior, visual input to the fish drives recorded motor activity which in turn affects the visual stimulus constituting a closed-loop feedback between the fish brain and its environment. We compared data from a closed-loop condition, where the fish actively maintain their position in the virtual environment, to a replay condition, where the same fish receives a replay of the closed-loop visual stimulus without real-time visual feedback (Fig 5A). The original work, [23], utilized the replay condition to identify neurons whose activity were strongly correlated either with the sensory input or motor output. Instead, here we use the replay condition as a strong control condition to reveal how neural dynamics are changed by the presence of sensory feedback rather than exafferent sensory input. Notably, one of the strengths of this setup is that the only information the fish received about oncoming flow was visual, i.e., there was no proprioceptive input as the fish was paralyzed.

**Fig 6:**
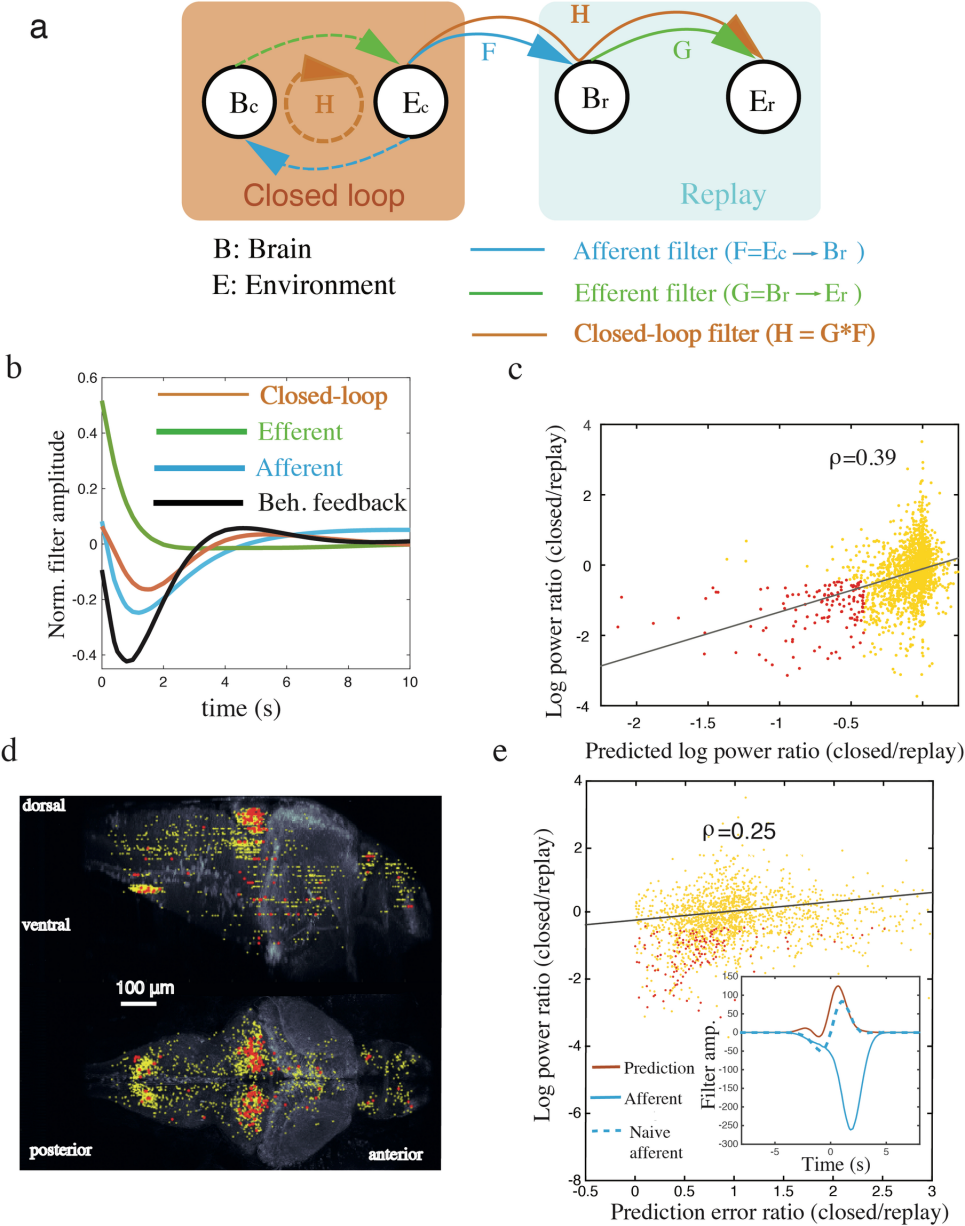
Closed-loop sensory feedback predicts suppression of neural fluctuations and correlations. **a** Schematic interactions of the brain (each neuron) and the environment (the swim power) are shown under the closed-loop (B_c_ and E_c_) and replay (B_r_ and E_r_) conditions. **b**, The afferent filter (F) and efferent filter (G) were estimated using the replay condition for each neuron by fitting linear filters (see Materials and Methods), whose population averages, after normalizing to peak amplitudes, are summarized. On average, the brain positively drove the environment, i.e. neural activity increased swim power (efferent filter G, green line) but the environment suppressed the brain, i.e., swim power tended to inhibit neural activity (afferent filter F, blue line). By combining these two effects, we found that self-feedback (H = F*G, orange line, closed-loop feedback) was negative. Behavioural feedback (E_c_ → E_r_, black line) is also strongly negative. **c**, These filters were then used to predict changes of neural fluctuations under the two conditions. The predicted changes in each neuron based on the filters exhibited strong correlation with the actual changes. Some neurons (top 10%, red dots) exhibited strong negative feedback and were suppressed under the closed-loop condition as predicted by our theory. **d,** The location of these neurons are overlaid with the morphology of a reference zebrafish, colors as in **c**. Top panel, side view; bottom panel, top view. Neurons that have strong negative feedback and reduced neural fluctuations were predominantly located in the cerebellum. **e**, The naive estimate of the afferent filter for each neuron in the closed-loop condition (quantified by the E_c_ → B_c_ filter, naive afferent) was qualitatively different from the afferent filter in the replay condition, see dashed and solid blue respectively (inset). However, the discrepancy was well explained by our theory that accounted for the closed-loop feedback effect (Inset, brown line; see Materials and Methods). The prediction error ratio ($\frac{R_{prediction}}{R_{filter}}$, see text) was correlated with the degree to which low-frequency power is suppressed during the closed-loop condition (yellow dots). Cells strongly stabilised by closed-loop feedback were explained well by the prediction (top 10%, red dots, as in **c**).

We found that neural activity was significantly different between the closed-loop and replay conditions despite identical sensory input, see Fig 5B. In particular, we found both the average magnitude of neural fluctuations and cross-correlations were suppressed in the closed-loop condition. While individual neurons were heterogeneous across the whole brain, on average low frequency (0.01–0.15 Hz) fluctuations were suppressed (p = 0.046, sign test) and neurons were decorrelated (p = 0.005, sign test) under the closed-loop condition compared to the replay condition (Fig 6A). The changes in the geometric mean of low frequency power and correlation were highly correlated across simultaneously recorded pairs of cells (r = 0.69, p<10^−10^, Spearman’s rank correlation), consistent with the hypothesis that both changes reflect the strength of sensory feedback. (see Fig 5C). The decorrelation effect was not an artifact of measurement noise, which may dominate correlation measures at high frequency, because the result was robust to the removal of low-level calcium activity by thresholding (see S3 Appendix).

The clearest difference between the closed-loop and replay conditions was the presence of sensory feedback suggesting that it plays a causal role. One possibility is that feedback increases the level of motor activity resulting in an increased efference copy signal which suppresses synchronous neural fluctuations [11,26]. However, while motor activity levels were higher in the closed-loop condition than in the replay condition, the trial-by-trial variation of neural fluctuations was not explained by the motor activity level. Specifically, increases in motor activity level were correlated with increases in low frequency power (r = 0.18, p < 10^−2^, Spearman’s rank correlation) and was not significantly correlated with changes in pairwise correlations between cells (r = 0.03, p > 0.5, Spearman’s rank correlation), see S3 Appendix.

We investigated if the strength of the sensory feedback could explain the observed changes in low-frequency neural fluctuations, as predicted by our theory. Specifically, we asked if the feedback mediated by the environment, estimated in the replay condition, predicted the degree to which each neuron is suppressed in the closed-loop condition relative to the replay condition.

To quantify the interactions between neurons and environmental variable we fitted the data with linear filters that when convolved with GCaMP fluorescence of individual neurons, *B*, best recover environmental variable, *E* (defined as activity of motor neurons which uniquely determines the visual stimulus), and vice versa, see Materials and Methods. Specifically, for each observed neuron, we computed a linear filter, *F*, that describes how the closed-loop swim power *E*_*c*_ (as quantified by the activity of motor neurons; a putative environmental variable) affects replay neural activity *B*_*r*_ i.e an *afferent filter* (Fig 6A, blue solid). We calculated linear filter, *G*, that describes how this neural activity *B*_*r*_ affects the replay swim power *E*_*r*_, i.e., an *efferent filter* (Fig 6A, green solid; see Materials and Methods). Note: we refer to the swimming power as the environmental variable because, in this experimental setup, visual stimulus is uniquely determined by a simple transformation of the motor nerve activity [23]. It is reasonable to assume that the same neural circuits characterized by these filters (Fig 6A orange solid) also operate in the closed-loop condition (Fig 6A orange dashed). Hence, we estimate the sensory feedback for each neuron (Fig 6A, orange dashed) as a filter, *H*, which is a convolution of these afferent, *F*, and efferent, *G*, filters (see Materials and Methods and Fig 6A, orange solid), i.e. *H* = F*G. Note: using the replay condition to fit these filters avoids the potential confound of calculating independent filters in the closed-loop condition. While these filters are neuron-dependent, the average peak-normalized filters showed clear net effects, see Fig 6B. On average, across cells, we found that the afferent filter (Fig 6B, blue line), was net negative, indicating that the swim-induced visual stimulus on average suppressed neural activity. The efferent filter (Fig 6B, green line), was strongly net positive, indicating that an increase in neural activity drove swim behavior. Finally, the convolution of these filters was on average also negative (Fig 6B, red line), peaking at about 1 s. This suggests, that on average, increases in neural activity self-suppressed after 1 s due to negative feedback. Notably, the negative sensory feedback interactions were also confirmed by a behavioral analysis, in which we calculated the linear filter that describes how *E*_*r*_ affects *E*_*c*_ (Fig 6B, black line), i.e. this represents a quantification of the *behavioural feedback*.

Next, we considered what the consequence of this negative feedback on each neuron's activity would be. Like the whisker system our theory suggests that fluctuations in neural activity should decrease if a cell receives negative feedback. If this is the case then we should be able to predict to what extent low frequency power is suppressed from the estimated strength of the feedback (see Materials and Methods for derivation of this prediction). We found that predicted degree to which a neuron’s activity was suppressed during the closed-loop condition relative to the replay condition was highly correlated with what was actually observed (r = 0.39, p < 10^−8^, Spearman’s rank correlation, see Fig 6C). To examine the role of ongoing cycles of feedback in these changes we estimated the degree to which a neuron’s activity would be suppressed by only a single cycle of feedback loop (see Materials and Methods). We found that for cells with the largest change in low frequency (i.e. top 10% of log power ratio, replay/closed) the mean square error of the full feedback prediction was significantly less than that of the single cycle prediction (p < 0.01, sign test; see Materials and Methods), indicating the importance of multiple cycles of feedback.

Although the fluctuations in activity in the majority of cells across the fish brain were suppressed by the presence of closed-loop behavior, the top 10 percentile of cells that were both strongly suppressed and strongly involved in the negative feedback were clustered in the cerebellum (Fig 6D), a brain area implicated in sensory-motor planning and coordination [30]. This supports the idea that the cerebellum plays a central role mediating negative closed-loop interaction between the brain and the environment by converting sensation into action in fish during optic flow stabilization [31].

Finally, we tested if the interaction between the brain and environment is shaped by closed-loop feedback. To examine this, we quantified the response of each neuron to sensory input in the closed-loop condition, i.e., by computing an afferent filter from the closed-loop data without accounting for the feedback loop, *a naive afferent filter* (see Fig 6A, blue dashed) and compared this with the afferent filter computed in the replay condition (see Fig 6A, blue solid). If feedback is weak the difference between the two should be small. These two filters were generally distinct in the observed neurons, but were particularly so for those cells that were strongly stabilized by feedback (Fig 6E; Inset). To test if the closed-loop interaction could explain this discrepancy, we theoretically predicted the naive afferent filter based on data from the replay condition, i.e., using both the afferent and efferent filters (see Materials and Methods). We found that, on average, this closed-loop effect could account for the discrepancy for the closed-loop stabilized cells (Fig 6E; Inset). To quantify this for each cell, we calculated the mean square error between the prediction and naive afferent filter (*R*_*prediction*_), and the mean square error between the afferent and naive afferent filter (*R*_*naive*_). The performance of the prediction is then quantified by the prediction error ratio ($\frac{R_{prediction}}{R_{naive}}$). This ratio was significantly less than one (median = 0.8, p < 10^−11^, sign test), indicating that the prediction was more accurate when accounting for the closed-loop effect. It was also positively correlated (r = 0.25, p<10^−13^, Spearman’s rank correlation) with the degree to which individual cells were stabilized in the closed-loop condition (Fig 6E). Altogether, these results indicate that neural dynamics, as well the relationship between sensory stimulus and behavior, not only depend on brain circuits, but are also dynamically shaped by the mutual interaction between the brain and the environment.

## Discussion

**Fig 7:**
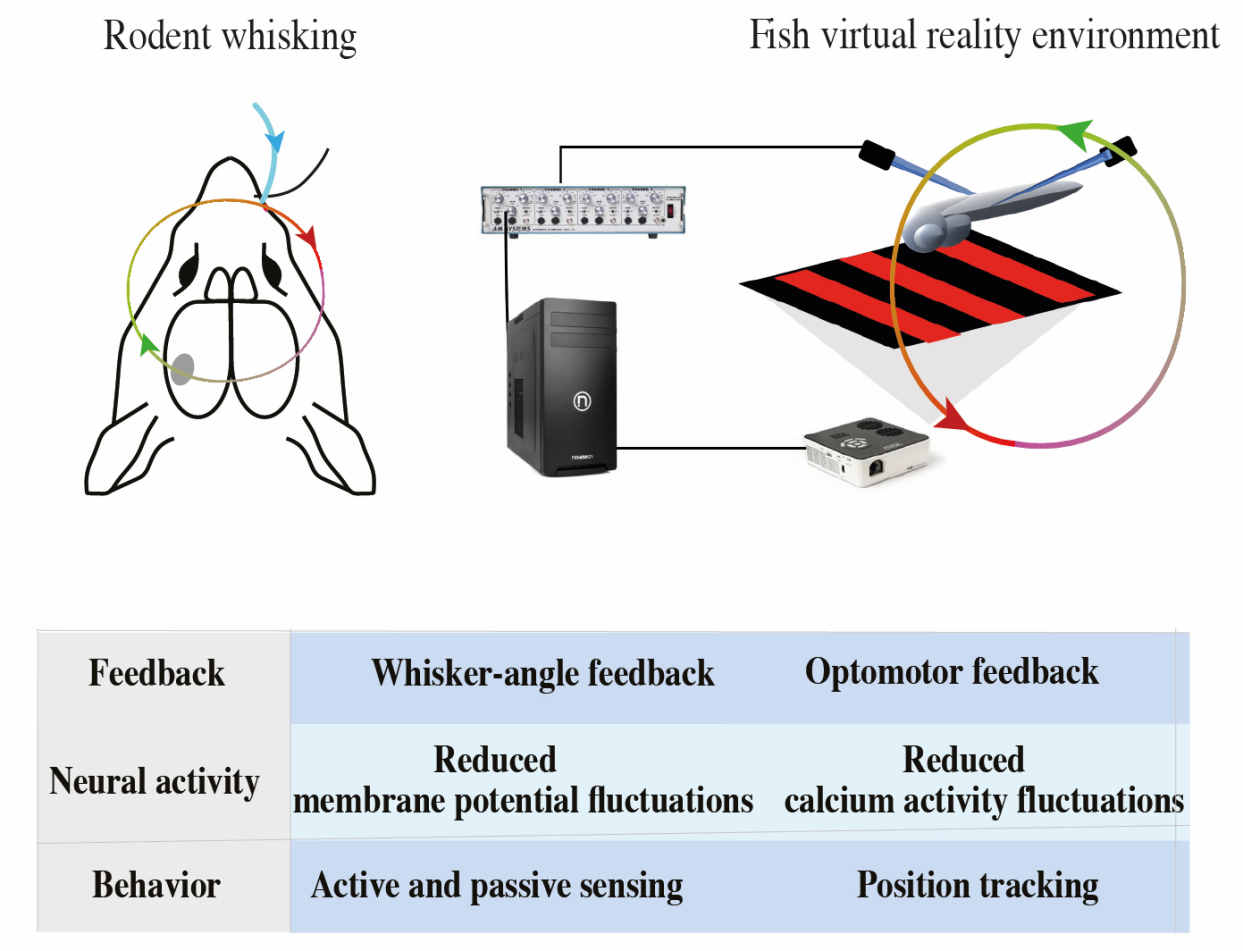
A summary of the experimental systems studied. Closed-loop sensory feedback (Top) explains neural activity in both systems, as summarized in the table (Bottom).

In this study we proposed the idea that negative closed-loop sensory feedback during active behavior reduces network gain, which in turn, suppresses synchronous neural fluctuations and modulates sensory responses. We supported this with modelling and data analysis in the whisker system and in a behaving zebrafish, see summary Fig 7. The formal component of our theory, i.e., that closed-loop sensory feedback can modulate a system's gain, is well documented in dynamical systems theory and control theory [32,33]. This gain control occurs even though the pathways mediating feedback are purely additive (c.f. Eqs 1 and 2; i.e., effectively repeated cycles of feedback accumulate over time and produce a multiplicative effect). Thus, a constitutively active closed-loop feedback that mediates action-perception cycles is essential for the form of gain control we propose. This means that discrete and intermittent involvement of reafferent input does not imply gain modulation. For example, the classical reafference principle explains neural responses by a one-time detection of the mismatch between an efference copy (predicted) and reafferent (actual) input [16]. However, this situation is likely an inaccurate idealization to describe the closed-loop systems studied here. For example, in the zebrafish system, swim bouts typically occur every 700 ms and this interval closely overlapped with the peak of the estimated sensory feedback interaction (Fig 6B). Hence, the neural responses in the fish experiment suggest a more dynamic system, where neural activity evoked by many cycles of action and sensation are continuously and mutually interacting.

The idea that closed-loop feedback is central to cognition is not new and has early precedents in behavioral psychology [19], resonate with a movement in embodied cognitive science [18,34,35] and has recently been proposed as concrete alternative to input/output conception of perceptual processing [17,36]. Our work shares the view of these proposals and provides a specific example where brain function is contingent on closed-loop interactions between brain/body/environment. Furthermore, we provided a mathematical model showing why neural dynamics underlying cognitive states cannot be recapitulated even if the sensory input during active behavior is identically repeated, i.e., a replay condition [37].

The presence of continuous negative closed-loop sensory feedback during active behavior is fundamental for our theory. In our rodent study we assumed negative closed-loop sensory feedback was mediated directly by a cortical-whisker circuit. However, our theory is agnostic to the detail of the neural implementation and several other schemes are possible (see S2 Appendix). This assumption is consistent with the idea that the barrel cortex comprises a nested set of servo control loops that regulate various aspects of whisker dynamics [22]. At the level of the whole vibrissa system multiple parallel and nested feedback loops both positive and negative most likely exist [22].

In zebrafish, the presence of negative feedback during swimming behavior is a priori necessary for optic-flow stabilization behavior because the fish must act in opposition to perceived optic flow in order to minimize horizontal displacement [38,39]. Interestingly, neurons that received strong negative feedback and were substantially stabilized were located in the cerebellum (Fig 6D). This is consistent with the theoretical viewpoint that the cerebellum is strongly involved in the action-perception cycle [40–42].

We suggest that closed-loop sensory feedback plays a major role in brain state control.

However, importantly, we do not propose this mechanism is mutually exclusive with other mechanisms, such as thalamo-cortical input [25] or neuromodulation [10,43,44] because brain state transitions also occur in the absence of sensory feedback e.g., the onset of running that does not change the visual input [3,45], during sleep [46,47], or by dissection of the sensory nerve [5,25]. Mechanisms underlying brain state transitions are likely to be redundant and occur even in the absence of mechanisms, such as thalamo-cortical input [25] or corollary discharge [26], albeit involving further delay (see S1). Such functional redundancy may help to maintain the stability of brain state [44,48,49]. Furthermore, the relative importance of internal and external mechanisms might adaptively change in an experience-dependent manner [50].

In the whisking model, we proposed that the regulation of cortical gain by closed-loop sensory feedback could explain enhanced active touch. Specifically, negative sensory feedback during whisking reproduces suppressed fluctuations and reduces responses to passive whisker stimulation (see Figs 2 and 3). Moreover, robust neural response to active touch events could be explained by the interruption of this feedback when the whisker is driven into an external object. These interruptions transiently release the cortex from a low gain state and enhancing sensory responses to salient sensory stimuli. This mechanism for active touch contrasts with the account of sensory processing suggested by the reafference principle [16], which postulates that motor efference is discounted from sensory input, allowing animals to sense exafferent signals (externally caused sensory input) without being confounded by the consequences of their own motor actions. In contrast, our theory suggests that the sensory system is insensitive to pure exafference during active sensing [4]; see Fig 3, but sensitive to the interruption of reafference which may allow animals to focus attention on the consequences of their own motor actions. This idea is supportive of other work that has cast doubt on the role of efference copy during active sensing [51]. This mechanism is also distinct from the most common form of predictive coding [52], where neural activity represents the error between the actual and the brain’s prediction of sensory input. Instead our suggestion could be viewed as a more specific form of predictive coding where neurons represent predictions errors about consequences of motor actions, in this sense it is closer to the idea of active inference [53,54].

While it is straightforward to generalize this sensory mechanism to other tactile systems, its implication for other modalities is less clear. However, in theory, closed-loop sensory feedback could be interrupted anywhere along the action-perception cycle, thus dynamically regulating neural gain. The timely interruption of this feedback, possibly related to transient freezing of behavior, could serve as a general mechanism for temporarily accentuating neural responses against a background of reduced noise. For example, closed-loop sensory feedback could be gated by the frequency of miniature eye movements [55] a hypothesis that complements a previous proposal suggesting such movements are under active closed-loop control [56]. Furthermore, cerebellum neurons, which are strongly involved in the sensory-motor cycle, could be suppressed in anticipation of salient sensory events by a relevant brain area, such as the reticular formation [31,57].

The importance of using naturalistic sensory stimuli to study and manipulate brain state dynamics is widely demonstrated [58]. However, an important prediction of our theory (Fig 4), supported by our experimental findings is that brain dynamics during active sensing cannot be fully recapitulated or re-encoded, even if the same sensory input is precisely recorded and replayed back into a passive brain. These results provide evidence that brain state during active behaviors can only be accurately understood by a quantitative account of ongoing brain-environment interactions [18].

## Material and methods

### A whisking model

To investigate the ‘in principle’ feedback between barrel cortex and whiskers we model a simple cortical circuit that interacts with a single whisker, see Fig 2A. Our cortical circuit comprises of *N* excitatory and *N* inhibitory neurons (i = 1…*N* are excitatory and *i* = *N*+1,…, 2*N* are inhibitory, *N* = 100) modeled as a linear dynamical system by,

$${\dot{x}}_{i}=-x_{i}+\sum_{j=1}^{2N}w_{i}{}_{j}x_{j}-a_{i}-w_{x}{}_{\theta }\theta_{p}+\xi_{i}+I,$$

which is numerically simulated by a Euler forward integration method with time-bin d*t* = 0.5 ms. Hereafter, all time derivatives are taken to represent single-step differences divided by *dt* (e.g. $\dot{x}(t)=\left[x(t+dt)-x(t)\right]/dt)$, but we omit the ms time unit. *w*_*ij*_ is the synaptic strength from neuron *j* to *i*, *a*_*i*_ is an adaptation current described below, *θ*_*p*_ is the whisker protraction angle interacting with neurons with weight *w*_*xθ*_ = 0.002, *I* is exafferent input that takes *I* = 0.035 upon whisker stimulation and otherwise zero, and *ξ*_*i*_ is independent white noise of unit variance added to each neuron. We interpret *x*_*i*_ as both the firing rate and membrane potential, assuming a roughly linear relationship between the two. Entries in the connectivity matrix are assigned as *w*_*ij*_ = *b*_*ij*_*J* + *b*′_*ij*_*g* for excitatory synapses (*j* = 1…, *N*) and *w*_*ij*_ = −*b*″_*ij*_*g* for inhibitory synapses (*j* = *N* + 1,…,2*N*), where *b*_*ij*_, *b*′_*ij*_, *b*″_*ij*_ are all random binary values that take *b*_0_ > 0 with probability *p* = 0.1 and 0 with probability 1 − *p*, respectively. The weights are scaled by $J=\frac{1}{pN}$ and $g=\frac{g_{0}}{\surd 2Np(1-p)}$, so that dynamics are insensitive to the parameter values of *p* and *N*. Note that the eigenvalue spectrum of the connectivity matrix *w*_*ij*_ is centered around *b*_0_ and spread with the radius *b*_0_*g*_0_ in the limit of large *N*. Hence, the network is excitation dominated. The variability of weight values across neurons is controlled by the magnitude *b*_0_*g*_0_ of the excitatory-inhibitory-balanced component and this variability is controlled by the parameter *g*_0_ = 0.05, which reproduces highly synchronized up/down-like fluctuations during the quiet state. To promote significant network fluctuations observed in the barrel cortex we scale of the connectivity matrix *b*_0_ such that the lead eigenvalue of this matrix is close to unity (≈ 0.975 and the dynamics are close to instability. We include an adaptation current that gives these fluctuations a low frequency (ca. 1 Hz) component modelling up/down-like oscillations [59–61] in the absence of neuron/whisker interactions. The adaptation current is integrated as

$$\dot{a_{i}}=-0.07a_{i}+0.008x_{i}$$

Over time, the adaptation variable slowly builds upon neural activity and suppresses neurons, resulting in the ca. 1-Hz oscillation. Consequently, in the absence of interactions with the whisker, implemented by setting *w*_*xθ*_ = 0, this simple network reproduces the power spectrum and cross-correlogram of neurons in the barrel cortex [5,6], see Fig 2B and 2C.

We model a simple flexible vibrissa as two hinged sections (with bending angle *θ*_*h*_) connected at the base (with protraction angle *θ*_*p*_ to the body) of unit length which are constrained by simple torsion springs with spring constant *k*_1_ and *k*_2_ respectively, see Fig 2A. We assume the whisker is light and frictionless and simulated it by numerically minimising the energy of the system,

$$E=k_{1}{\left(\theta_{p}-\theta_{eq}\right)}^{2}+\frac{k_{2}}{2}\theta_{h}^{2},$$

where *θ*_*eq*_ equilibrium value of the base spring. Here, only the ratio *k*_1_/*k*_2_ is important for the results and, without losing generality, we set *k*_1_ = 1. The central hinge spring has an equilibrium value of zero angular displacement and thus tends to align both sections. Whisking is driven both by the cortex and a central a pattern generator (CPG) [62]. Specifically, the equilibrium value of the base spring, *θ*_*eq*_ is set as,

$$\dot{\theta_{eq}}=-0.93\theta_{eq}+\frac{w_{\theta x}}{N}\overset{N}{\underset{i=1}{\sum }}x_{i}+u_{,}$$

where the second term on the right-hand side is the sum of activity in the cortical excitatory population and the third term is the external CPG activity. Here *u* is modeled as simple stochastic oscillator, given by

$$\dot{u}=-.98u+2\pi F_{whisk}v+\xi_{u}$$

$$\dot{v}=-.98v-2\pi F_{whisk}u+\xi_{v},$$

where *F*_*whisk*_ = 10Hz is the frequency of the oscillator and *ξ*_*u*_, *ξ*_*v*_ are independent Gaussian white noise. *w*_*θx*_ = 0.085 describes the relative strength of the cortex versus the CPG in driving the whisker variable. With this parameter, the whisker is mainly driven by the CPG and is only modulated by cortical activity. In this model, most excitatory neurons respond to whisker retraction and drive whisker protraction. Adding a separate counterpart population that responds to whisker protraction and drives whisker retraction in a similar manner does not change the model’s behavior.

We simulate a passive deflection of the whisker by a brief injection of input of *I* = 0.035 to the cortical neurons for c.a 25 ms. The magnitude of this input approximately matches the evoked change over the standard deviation of the membrane potential ($\Delta V_{m}/\sigma_{V_{m}}$) in response to magnetic whisker deflection during the whisking condition [5].

Contact events are simulated by simulating a horizontal solid wall is placed above the whisker (1 unit length away). To simulate contact with the wall we solve the energy equation subject to the length constraint in the vertical direction,

$$sin(\theta_{p})+sin(\theta_{p}-\theta_{h})<1.$$

Thus, as the whisker collides with the wall it deforms accordingly, see Fig 2A. By adjusting the relative stiffness of each torsion spring (i.e. *k*_2_/*k*_1_), we can control the degree to which the protraction angle is affected by contact events, e.g., if the whisker is very flexible, the protraction angle will change continuously, despite contact of the tip. During contact we also inject an input (*I* = 0.035) to the cortical neurons for the duration of the contact event, but for no longer than 25 ms to simulate contact-detection signal that results from the stereotypical response of pressure sensitive cells in the trigeminal ganglion [27]. The model was run for 200 s in the closed loop, open-loop, and sustained period of active touch to calculate all quantitative measures.

### Quantifying signal-to-noise in the whisking model

To quantify the discriminability of whisker contact events we calculated an information theoretic measure of generalized signal-to-noise-ratio. Specifically, we calculated the Chernoff distance [63–65] between probability distributions, *p*_1_(*x*) and *p*_0_(*x*), in the presence or absence of a sensory event, respectively. Specifically, this measure

$$\Psi (p_{1}∥p_{0})\equiv -\underset{0<\lambda <1}{min}\log\;\int {p_{1}}^{\lambda }(x){p_{0}}^{1-\lambda }(x)dx$$

summarises the detectability of whisker stimulation based on population responses and, unlike a naive calculation of signal-to-noise ratio, is applicable even when *p*_1_(*x*) and *p*_0_(*x*) are very different distributions. For our model, the probability distribution for each condition is well described by a Gaussian distribution,

$$p_{0/1}(x)=|2\pi C_{0/1}{|}^{-1/2}exp(\frac{-1}{2}(x-\mu_{0/1}){C_{0/1}}^{-1}(x-\mu_{0/1})),$$

where *C*_0/1_ and *μ*_0/1_ are covariance matrix and vector of means, respectively, in the presence (with subscript 1) or absence (with subscript 0) of a sensory event. By substituting this into the expression for Chernoff distance and employing the Gaussian integral identity and expressing the Chernoff distance in terms of *C*_0_, *C*_1_, and *μ*_0_, *μ*_1_, we calculate the covariance and mean between a small number of neurons (here three), randomly selected from the cortical network described above. We calculate covariance’s across ensembles of 500 networks every 10 ms for a period of 1 s, starting at the onset of the sensory event. Minimization with respect to *λ* is computed numerically.

### Zebrafish experimental data

In a transgenic fish expressing the calcium indicator GCaMP2 brain-wide calcium activity was monitored using a two-photon microscope to scan single planes in the brain. We analyzed the calcium signal (ΔF/F) at various sample frequencies (ca. 2−3 Hz) across 1908 cells in 32 fish, see [23] and electrical recordings of swim power. We analyzed data taken from a 6-min recording of 1−6 prominent calcium sources per fish, putative neurons, across 600 trials. In the first 3 min, the fish performed the closed-loop optomotor behavior. For the subsequent 3 min, each fish was presented with the stimulus received in the closed-loop stimulus which is a repeat of what the animal experienced in the previous 3 min, the replay condition.

In the original study, the gain (i.e., the multiplicative factor between fictive swim power, and the speed of visual feedback) was alternated between a high and low gain condition every 30 s. This gain alternating protocol is not relevant to the current study. To reduce this variability in data, we subtracted the mean activity level in each gain setting in our analysis (from both brain and behavior variables). Notably, our main results were qualitatively the same, even without such subtraction of the means.

### Zebrafish data analysis

We distinguish variables in the closed loop condition (*B*_*c*_ and *E*_*c*_) and replay condition (*B*_*r*_ and *E*_*r*_), see Fig 5B. Specifically, we assume that the closed-loop dynamics in the frequency domain are described by the following equations,

$$B_{c}(\omega )=F(\omega )E_{c}(\omega )+R_{B_{c}}(\omega )$$ (4)

$$E_{c}(\omega )=G(\omega )B_{c}(\omega )$$

where *F*(*ω*) is an afferent filter describing the interaction from the environment to the brain (i.e., the *E*_*c*_ → *B*_*c*_ filter, see Fig 5B dashed blue arrow) and *G*(*ω*) is an efferent filter from the brain to the environment (i.e., the *B*_*c*_ → *E*_*c*_ filter, see Fig 5B dashed green arrow), respectively, and $R_{B_{c}}(\omega )$ is the residual inputs not accounted of by the filters. Note: we have assumed that the noise on the environment is negligible, this is a reasonable assumption given that visual flow is directly modulated by motor nerve activity. Similarly, we also write the replay dynamics in the frequency domain as,

$$B_{r}(\omega )=F(\omega )E_{c}(\omega )+R_{B_{r}}(\omega )$$ (5)

$$E_{r}(\omega )=G(\omega )B_{r}(\omega ).$$

In the replay condition, neurons are driven by the recorded visual stimulus in the closed-loop condition, which is determined by fish’s motor activity in the closed-loop condition *E*_*c*_. Note: we have made the assumption that *F*(*ω*) and *G*(*ω*) are the same filter in the both conditions (i.e., the interactions with the same color in Fig 6A have the same property) because the sensory and motor circuits in the brain remain the same between the conditions.

We use Eq 5 in the replay condition to fit the linear filters *F*(*ω*) and *G*(*ω*) because the computation would be more involved in the closed-loop condition than the replay condition. We first calculate linear filter *F* (Fig 6A, solid blue arrow) that minimizes the mean square error between the observed variable *B*_*r*_ and the convolution *F* * *E*_*c*_ over time. Next, we determine *G*(*t*) by first calculating the residual variability of neural activity in the replay condition that cannot be accounted for by the closed-loop environment, i.e., $R_{B_{r}}(\omega )=B_{r}(\omega )-F(\omega )E_{c}(\omega )$ and subsequently calculating how $R_{B_{r}}$ drives the environment in the replay condition *E*_*r*_, effectively determining the *B*_*r*_ → *E*_*r*_ interaction (Fig 6A, solid green arrow). The filters were constrained as a superposition of Laguere functions. We use Laguere functions up to the order that best satisfied the Akaike Information Criterion [66]. Almost all filters had an order that was mid-range between 1 and 15. The *E*_*c*_ → *B*_*r*_ → *E*_*r*_ interaction (Fig 6A solid orange arrow) is then straightforwardly computed by the convolution of both filters, *H*(*ω*) = *F*(*ω*)*G*(*ω*). Based on the assumption that the filters are the same in the two conditions, we assume that self-feedback in the closed-loop condition (Fig 6A, dashed orange arrow) is the same as *H*(*ω*).

In our investigation, we calculated the ratio of the low frequency power of neural fluctuations between the closed-loop and replay conditions. We then compare this empirical ratio with the theoretically expected ratio based on the estimated filters. To derive this theoretically expected ratio, we write the dynamics of neural activity in the closed- and replay conditions in the frequency domain as,

$$\begin{array}{l} Closed‑loop:\;B_{c}(\omega )=H(\omega )B_{c}(\omega )+R_{B_{c}}(\omega )={\left(1-H(\omega )\right)}^{-1}R_{B_{c}}(\omega )\\ Replay:\;B_{r}(\omega )=H(\omega )B_{c}(\omega )+R_{B_{r}}(\omega ), \end{array}$$

where *H*(*ω*) = *F*(*ω*)*G*(*ω*) is the estimated combined filter in the frequency domain and we assume the noise in the closed- and replay conditions have the same power spectrum, i.e., ${R_{B_{c}}(\omega )}^{2}={R_{B_{r}}(\omega )}^{2}$. The ratio of the power between each condition is then,

$$\frac{{B_{c}(\omega )}^{2}}{{B_{r}(\omega )}^{2}}=\frac{1}{{H(\omega )}^{2}+1-{H(\omega )}^{2}}.$$

We also investigated the effect of accumulative cycles of feedback on brain dynamics by comparing the full closed-loop effect with a control effect that includes only one-time feedback. Namely, we can expand the contribution of each cycle in a geometric series as

$$B_{c}(\omega )={\left(1-H(\omega )\right)}^{-1}R_{B_{c}}(\omega )=(1+H(\omega )+H^{2}(\omega )+H^{3}(\omega )\cdots )R_{B_{c}}(\omega )$$

where the O(*H*^*n*^) term in the above Taylor expansion describes the effect from signal propagation along the feedback loop for *n* times. By neglecting the contributions with *n*>1, we can write the effect of a single cycle of feedback effect as,

$$B_{1}(\omega )=(1+H(\omega ))R_{B_{c}}(\omega ).$$

This yields an alternative expression for the ratio of the power between each condition that only includes one-time effect of feedback as,

$$\frac{{B_{1}(\omega )}^{2}}{{B_{r}(\omega )}^{2}}=\frac{1}{{H(\omega )}^{2}+1+{H(\omega )}^{-2}}.$$

To further investigate how the effective interaction between the brain and the environment depends on the closed-loop feedback, we compare *E*_*c*_ → *B*_*r*_ filter in the replay condition and the *E*_*c*_ → *B*_*c*_ filter in the closed-loop condition naively computed by neglecting closed-loop effects. Notably, the naïve *E*_*c*_ → *B*_*c*_ filter in the closed-loop condition generally has an acausal component, because the brain *B*_*c*_ and the environment *E*_*c*_ are mutually interacting (see below). Thus to calculate these filters we use Hermite rather than the Laguere functions to capture the acausal (t<0) side of the filter. To quantify the difference between these filters, using Eq 4, we write

$$E_{c}(\omega )={\left(1-H(\omega )\right)}^{-1}(G(\omega )R_{B_{c}}(\omega )),$$

and thus the naïve *E*_*c*_ → *B*_*c*_ filter in the closed-loop condition is

$$\frac{B_{c}(\omega ){E_{c}}^{*}(\omega )}{E_{c}(\omega ){E_{c}}^{*}(\omega )}=F(\omega )+{\left(\frac{E_{c}(\omega )R_{B_{c}}^{*}(\omega )}{E_{c}(\omega ){E_{c}}^{*}(\omega )}\right)}^{*}=F(\omega )+{\left(\frac{G(\omega )}{1-H(\omega )}\right)}^{*}\frac{{\left|R_{B_{c}}(\omega )\right|}^{2}}{{|E}_{c}{\left(\omega )\right|}^{2}}$$

where * describes complex conjugate. Hence, this filter is different from the corresponding filter *F*(*ω*) in the replay condition by the second term. To predict the second term without knowing *R*_*Bc*_, we again assume ${\left|{R_{B}}_{c}(\omega )\right|}^{2}\approx {\left|{R_{B}}_{r}(\omega )\right|}^{2}$, where the latter spectrum is based on the residual $R_{B_{r}}$ computed in the replay condition.

## Supporting information

S1 Appendix Analaysis of supplementary whisker data. (DOCX)

S2 Appendix Alternate schemes for sensory feedback in the whisker system. (DOCX)

S3 Appendix Supplementary fish data analysis. (DOCX)
