# Supplementary material for: A theory of how active behavior stabilises neural activity: Neural gain modulation by closed-loop environmental feedback
Source: PLoS Comput Biol. 2018 Jan 17;14(1):e1005926. doi: 10.1371/journal.pcbi.1005926 (PMC5809098; doi:10.1371/journal.pcbi.1005926)
Supplement: S1 Appendix — (DOCX) [file pcbi.1005926.s001.docx]

**S1 Supplementary whisker data**

We analyze if sensory input through infraorbital nerve (ION) plays a role in coordinating whisking behavior, thalamic spiking activity, and cortical local field potential (LFP). Previous results based on simultaneous recording from whisker, thalamus, and cortex exhibited that thalamic spiking rate increased and low frequency power of cortical LFP decreased during whisking behavior. Here, we reanalyze this data set and quantify cross-correlation functions between (1) 5-20 Hz power of the whisker position, denoted by *Whisker*; (2) thalamic spiking rate computed with 20 ms averaging window, denoted by *Thalamus*; (3) and 1-20 Hz cortical LFP power, denoted by *Cortex*, recorded from ION-intact animals (n=22) and ION-cut animals (n=19). Raw recordings and the three processed traces are shown in Fig. S1A for an example animal. We chose the 1-20 Hz range for the analysis of cortical LFP power because notable brain-state-dependent changes were previously observed in this range [[1]](https://paperpile.com/c/ymIcYy/vtoyO). The spectrogram was computed using 2 s window to reliably estimate the predominant 1Hz power in cortical LFP and the window was gradually shifted in 20 ms steps.

Next, we computed cross-correlation functions between these 3 quantities: *Whisker*-*Thalamus*, *Whisker*-*Cortex*, and *Thalamus*-*Cortex*. While the resulting cross-correlation functions were noisy in each animal, a mean cross-correlation function averaged over each animal group exhibited clear common properties. In both ION-intact and ION-cut animals, whisking behavior lead correlated increase in the thalamic activity and decrease in the cortical slow oscillations. Consistent with this result, the thalamic activity was negatively correlated with the low-frequency cortical LFP fluctuations (Fig. S1B).

Notably, the position of the mean cross-correlation peaks was significantly shifted in ION-cut animals relative to the ION-intact animals (Fig. S1B). Specifically, the peak of the *Whisker*-*Thalamus* cross-correlation was delayed for 400 ms (p=0.02, bootstrap test) and the peak of *Whisker*-*Cortex* cross-correlation was delayed for 200 ms (p=0.03, bootstrap test) in ION-cut animals. However, the temporal relationship between the thalamic spiking activity and the low-frequency cortical LFP power was not significantly altered as assessed by the *Thalamus*-*Cortex* correlation function (p>0.05, bootstrap test). The bootstrap statistics were computed by randomly resampling animals from the two groups, assuming a null hypothesis that the two animal groups are the same (see, the inset panels for the bootstrap statistics about the difference of the cross-correlation peak locations).

These analyses suggest that the brain state transition was delayed after a whisking onset in ION-cut animals relative to ION-intact animals. Thus, while sensory input is not necessary for the brain state transition, it was necessary for inducing short-latency brain state transitions.


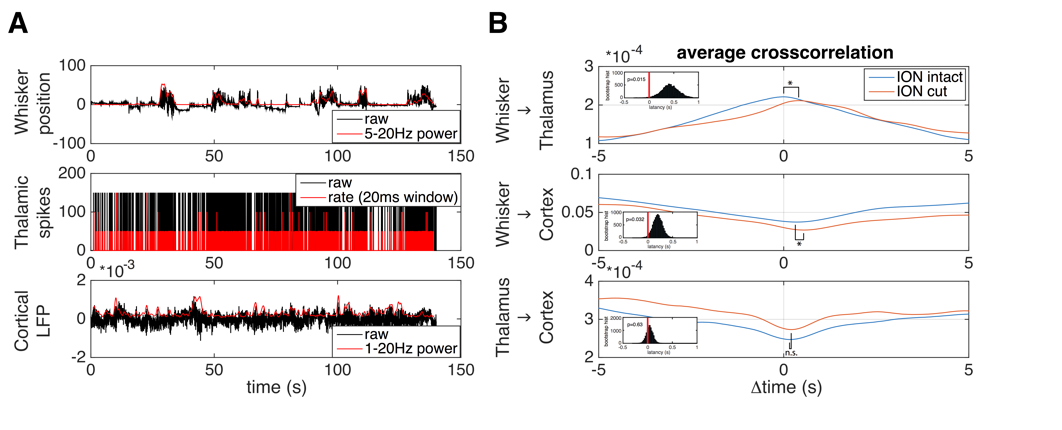


**Fig. S1 |** (**A**) A simultaneous recording of whisker position (Top), thalamic spikes (Middle), and cortical LFP (Bottom) in an example animal [2]. Based on these raw traces (black), brain-state-relevant quantities (red) are computed and shown in each panel: 5-20 Hz power of the whisker position (Top), thalamic spiking rate (Middle), 1-20 Hz power of the cortical LFP (Bottom). (**B**) A cross-correlation function between *Whisker* and *Thalamus* (Top), *Whisker* and *Cortex* (Middle), and *Thalamus* and *Cortex* (Bottom) for ION-intact animals (blue) and ION-cut animals (red), where the inset panels show the bootstrap statistics about the difference of the cross-correlation peak locations. The *Whisker*-*Thalamus* and the *Whisker*-*Cortex* correlation functions were significantly shifted by the ION cut.

1. [Poulet JFA, Petersen CCH. Internal brain state regulates membrane potential synchrony in barrel cortex of behaving mice. Nature. 2008;454: 881–885.](http://paperpile.com/b/5tq7Aa/NKxO)

2. [Poulet JFA, Fernandez LMJ, Crochet S, Petersen CCH. Thalamic control of cortical states. Nat Neurosci. 2012;15: 370–372.](http://paperpile.com/b/5tq7Aa/d0IJW)
