# Supplementary material for: A theory of how active behavior stabilises neural activity: Neural gain modulation by closed-loop environmental feedback
Source: PLoS Comput Biol. 2018 Jan 17;14(1):e1005926. doi: 10.1371/journal.pcbi.1005926 (PMC5809098; doi:10.1371/journal.pcbi.1005926)
Supplement: S2 Appendix — (DOCX) [file pcbi.1005926.s002.docx]

**S2 Alternate schemes for sensory feedback in the whisker system**

A


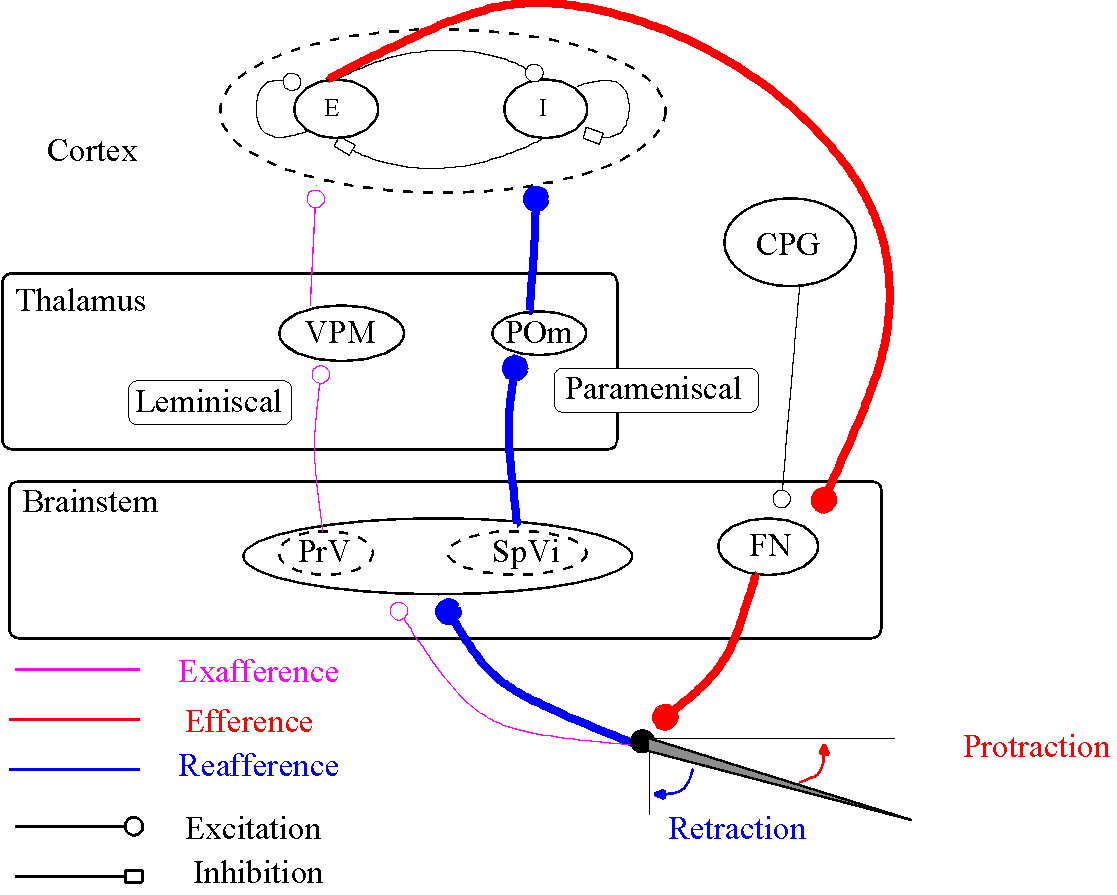


B


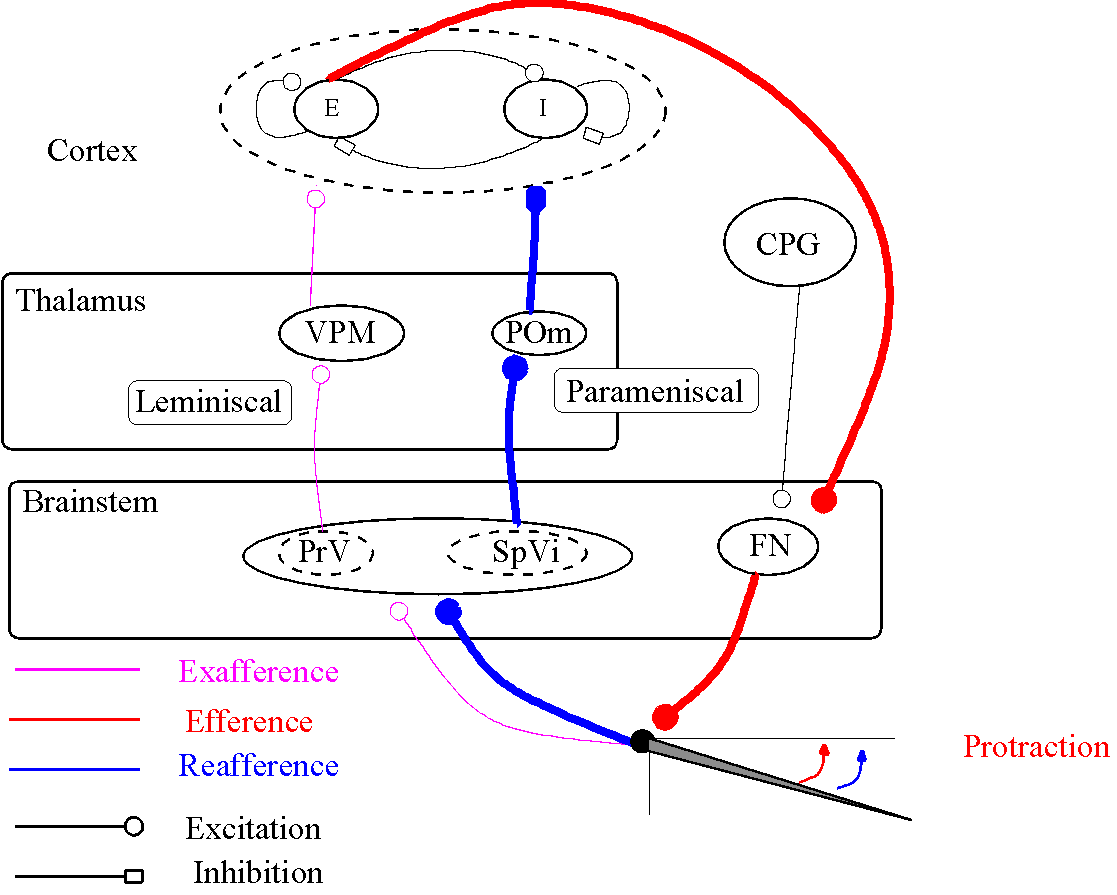


C


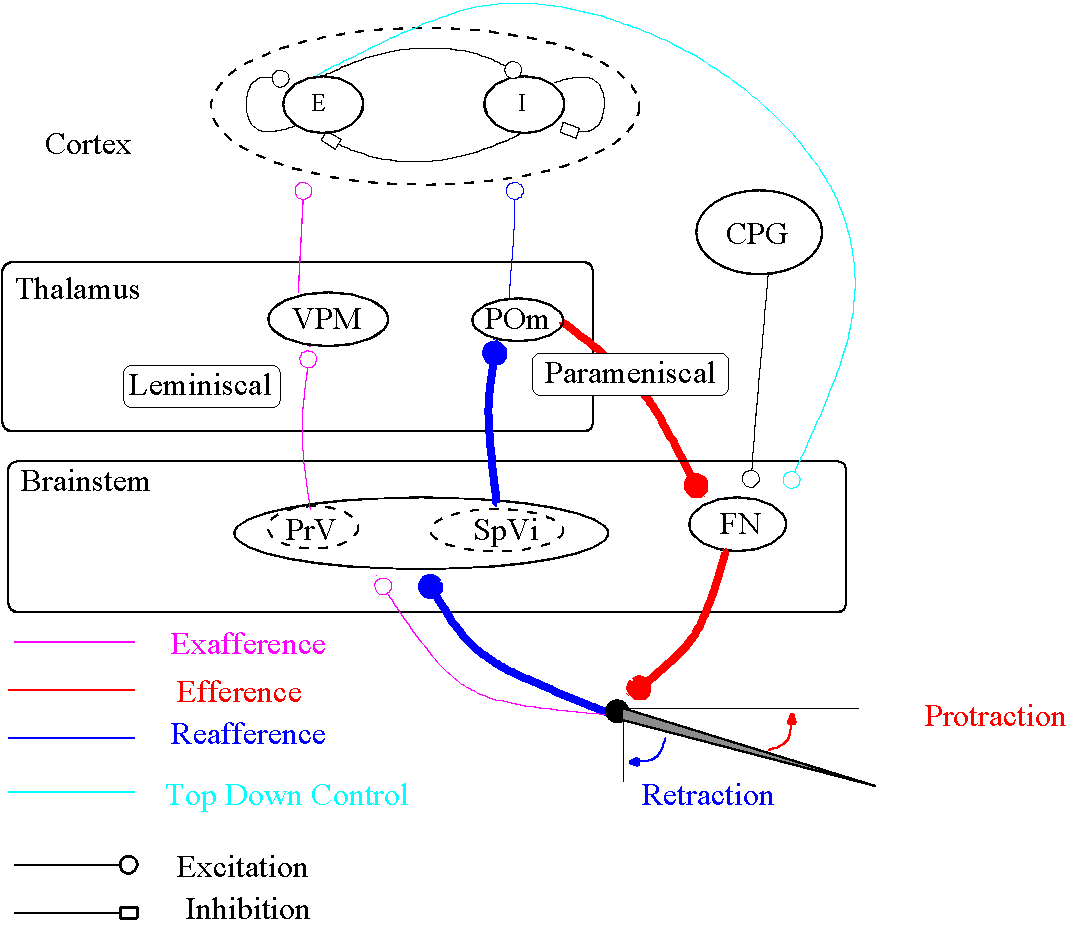


**Fig. S2**: Three models for whisker circuits mediating a negative closed-loop sensory feedback. (**A**) Net activation of the modeled cortical population drives neurons in the facial nucleus (FN) to drive whisker protraction. This in turn reduces excitatory sensory input to the modeled cortical population because this is driven by retraction. Here negative feedback is mediated implicitly at the periphery. (**B**) Protraction information could be conveyed along the full pathway but net inhibitory input to the modeled cortical population result because POm inhibits the cortex. In either model circuit, the initial activation of cortical neurons causes subsequent suppression of their activity by feedback through the whisker circuit, constituting a negative sensory feedback loop. These two hypotheses are testable but not necessarily mutually exclusive. (**C**) An alternative hypothesis is the whisker feedback is completed in the brain stem and changes in cortical activity are driven by activity changes in the thalamus.

**Fig. S2**: Three models for whisker circuits mediating a negative closed-loop sensory feedback. (**A**) Net activation of the modeled cortical population drives neurons in the facial nucleus (FN) to drive whisker protraction. This in turn reduces excitatory sensory input to the modeled cortical population because this is driven by retraction. Here negative feedback is mediated implicitly at the periphery. (**B**) Protraction information could be conveyed along the full pathway but net inhibitory input to the modeled cortical population result because POm inhibits the cortex. In either model circuit, the initial activation of cortical neurons causes subsequent suppression of their activity by feedback through the whisker circuit, constituting a negative sensory feedback loop. These two hypotheses are testable but not necessarily mutually exclusive. (**C**) An alternative hypothesis is the whisker feedback is completed in the brain stem and changes in cortical activity are driven by activity changes in the thalamus.

Our whisker model remains abstract in terms of known vibrissa system anatomy and, in particular, the relay stations between the cortex and a whisker. The exact concordance of the model with known vibrissa system anatomy is beyond the scope of this paper, but we provide a more detailed to demonstrate a possible anatomical explanation of our model and provide a means for the research community to experimentally examine closed-loop sensory feedback in specific biological circuits.

In our model (Fig. S2A), we assume that projections between regions are largely excitatory (c.f.[[1]](https://paperpile.com/c/5tq7Aa/XLTbZ)). Importantly, we distinguish two subcortical pathways that signal afferent input to cortical neurons - one for transmitting reafferent input and the other for transmitting exafferent input. This distinction could reflect the separation between a parameniscal pathway i.e., via thalamic POm, conveying reafferent signals, and a lemniscal pathway, i.e., via thalamic VPM, conveying exafferent input [[2,3]](https://paperpile.com/c/5tq7Aa/pPnbD+xgY1V)). Accordingly, we modeled exafferent input to cortical neurons by using a stereotypical pulse upon each whisker contact and brief deflection, and reafferent input proportional to whisker angle reflecting motor efference [[4]](https://paperpile.com/c/5tq7Aa/iuN4r). Regardless of how the properties of reaffererent input - whisking phase, absolute position, or their temporal derivatives - are encoded by the pathway they do not change the main conclusion of our model as long as the closed-loop sensory feedback constitutes net negative feedback.

In agreement with the anatomy, we assume that cortically generated motor signals modulate whisking behavior by acting on the facial nucleus (FN) [[1]](https://paperpile.com/c/5tq7Aa/XLTbZ). Because whisking behavior persists after sensory denervation [[5]](https://paperpile.com/c/5tq7Aa/PqJmK) , cortical ablation [[6]](https://paperpile.com/c/5tq7Aa/dveaU), or decerebration[[7]](https://paperpile.com/c/5tq7Aa/bucqI), we explicitly modeled a central pattern generator (CPG) that autonomously generates whisking patterns locating exogenous to the cortical-whisker loop [[8]](https://paperpile.com/c/5tq7Aa/rstQk). Thus, the FN receives input from both the cortical population and CPG and moves the whisker in the reafference model.

We cannot rule out other biological pathways for negative closed-loop sensory feedback. For example, negative feedback can also be mediated by dominant cortical inhibition to the modeled population of cortical neurons (Fig. S2B). In agreement, thalamocortical connections strongly innervate fast spiking neurons and consequently implement strong feedforward inhibition to the cortex[[9]](https://paperpile.com/c/5tq7Aa/jlDx4). Other potential models arise from heterogeneity in cortical populations. For example, negative closed-loop sensory feedback can be mediated by neurons in the barrel cortex that directly drive whisker retraction with extremely short latencies [[10]](https://paperpile.com/c/5tq7Aa/neYVr).

Alternatively, the dominant negative feedback loop could be subcortcial, Fig. S2C. Here the dynamics of the cortex only indirectly reflects the stabilization of the thalamus. This scheme is also consistent with reduced thalamic activity during whisking [[11]](https://paperpile.com/c/5tq7Aa/d0IJW). It is important to note that this implementation also fundamentally relies on stabilisation of neuronal activity by negative closed-loop sensory feedback.

At the level of the whole vibrissa system, there are likely multiple parallel and nested feedback loops, both positive and negative [[1]](https://paperpile.com/c/5tq7Aa/XLTbZ). However, we assume that the overall or net feedback mediated by the cortical-whisker circuit during corresponding behavior is negative in sign which we empirically demonstrate the presence of negative closed-loop sensory feedback in zebrafish active sensing. These models are experimentally testable. For example, a group of neurons that encode aspects of reafferent input, such as whisker protraction, could be genetically labeled and used for anatomical tracing studies. We can moreover study the physiological role of these neurons by optogenetically silencing them during active whisking and study how brain state as well as the animal’s behavior may be altered. This specific neuronal population could also be optogenetically activated and the ensuing behaviors and changes in vibrissae information processing pathways studied.

1. [Ahissar E, Kleinfeld D. Closed-loop neuronal computations: focus on vibrissa somatosensation in rat. Cereb Cortex. 2003;13: 53–62.](http://paperpile.com/b/5tq7Aa/XLTbZ)

2. [Pierret T, Lavallée P, Deschênes M. Parallel streams for the relay of vibrissal information through thalamic barreloids. J Neurosci. 2000;20: 7455–7462.](http://paperpile.com/b/5tq7Aa/pPnbD)

3. [Urbain N, Salin PA, Libourel P-A, Comte J-C, Gentet LJ, Petersen CCH. Whisking-Related Changes in Neuronal Firing and Membrane Potential Dynamics in the Somatosensory Thalamus of Awake Mice. Cell Rep. 2015;13: 647–656.](http://paperpile.com/b/5tq7Aa/xgY1V)

4. [Szwed M, Bagdasarian K, Ahissar E. Encoding of vibrissal active touch. Neuron. 2003;40: 621–630.](http://paperpile.com/b/5tq7Aa/iuN4r)

5. [Welker WI. Analysis of Sniffing of the Albino Rat 1). Behaviour. 1964;22: 223–244.](http://paperpile.com/b/5tq7Aa/PqJmK)

6. [Semba K, Komisaruk BR. Neural substrates of two different rhythmical vibrissal movements in the rat. Neuroscience. 1984;12: 761–774.](http://paperpile.com/b/5tq7Aa/dveaU)

7. [Lovick TA. The behavioural repertoire of precollicular decerebrate rats. J Physiol. 1972;226: 4P–6P.](http://paperpile.com/b/5tq7Aa/bucqI)

8. [Hill DN, Curtis JC, Moore JD, Kleinfeld D. Primary Motor Cortex Reports Efferent Control of Vibrissa Motion on Multiple Timescales. Neuron. 2011;72: 344–356.](http://paperpile.com/b/5tq7Aa/rstQk)

9. [Bruno RM, Simons DJ. Feedforward mechanisms of excitatory and inhibitory cortical receptive fields. J Neurosci. 2002;22: 10966–10975.](http://paperpile.com/b/5tq7Aa/jlDx4)

10. [Matyas F, Sreenivasan V, Marbach F, Wacongne C, Barsy B, Mateo C, et al. Motor control by sensory cortex. Science. 2010;330: 1240–1243.](http://paperpile.com/b/5tq7Aa/neYVr)

11. [Poulet JFA, Fernandez LMJ, Crochet S, Petersen CCH. Thalamic control of cortical states. Nat Neurosci. 2012;15: 370–372.](http://paperpile.com/b/5tq7Aa/d0IJW)
