# Supplementary material for: A theory of how active behavior stabilises neural activity: Neural gain modulation by closed-loop environmental feedback
Source: PLoS Comput Biol. 2018 Jan 17;14(1):e1005926. doi: 10.1371/journal.pcbi.1005926 (PMC5809098; doi:10.1371/journal.pcbi.1005926)
Supplement: S3 Appendix — (DOCX) [file pcbi.1005926.s003.docx]

**S3 Supplementary fish data**


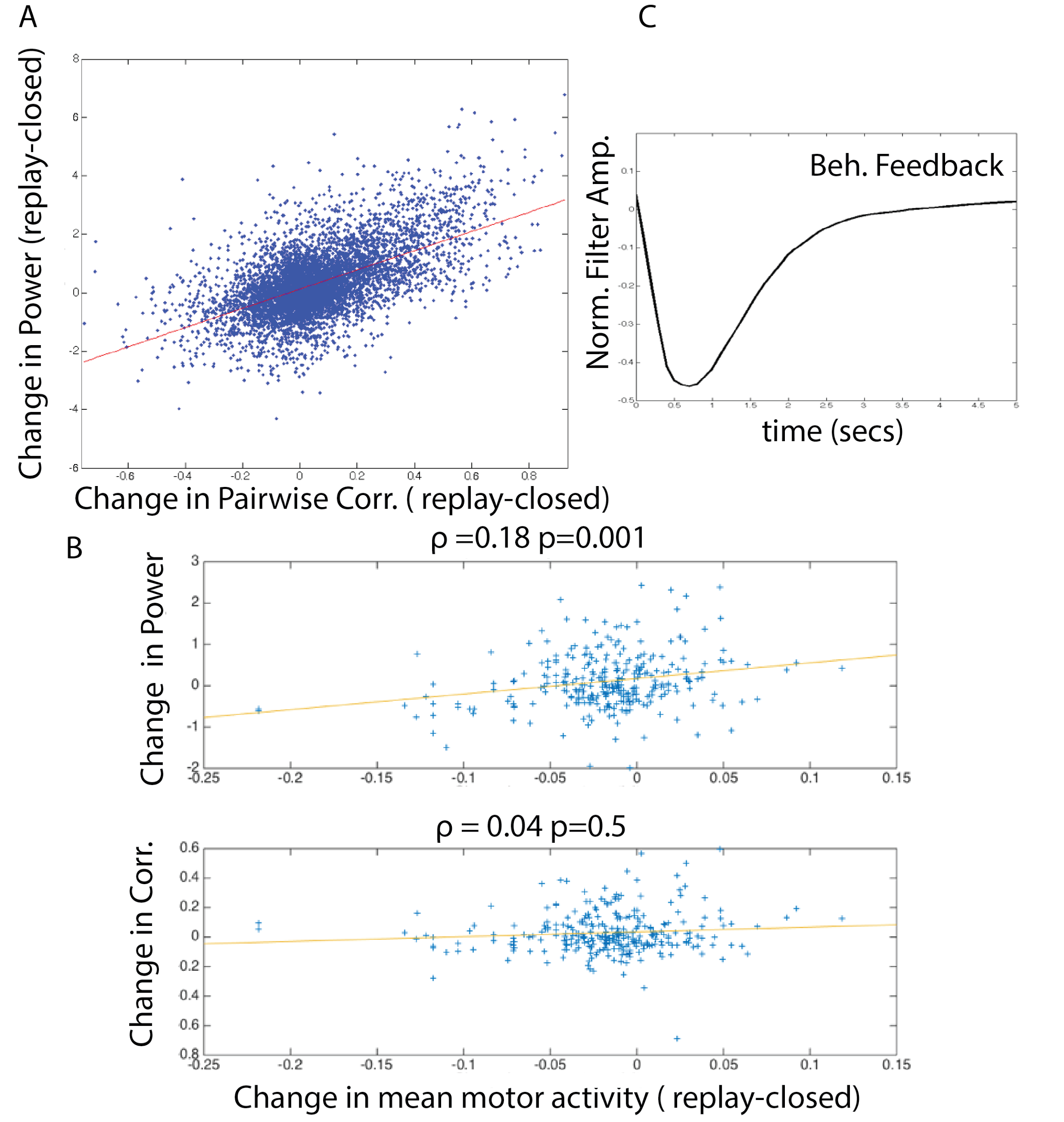


**Fig. S3 |** (**A**) The decorrelation effect is not an artifact of measurement noise. Changes in pairwise correlations (replay-closed and change in log low frequency power (replay-closed, mean over interval [0.01 0.15] Hz) were highly correlated in the recorded neurons even when the calcium traces (both cells and motor neurons) were thresholded (the threshold was equal to the mean plus one standard deviation of the calcium signal measured over both replay and closed loop conditions) (Spearman’s rank correlation ρ=0.57, p<10^-8^). (**B**) The increase of motor activity in the closed-loop condition does not easily explain reduction in neural fluctuations and correlation. (**B**,top): Changes in mean motor activity (replay-closed) and change in log low frequency power (as in **A**), averaged over all cells within a given trial are positively correlated (r=0.18, p<10^-2^, Spearman’s rank correlation). Specifically, on a cell-by-cell basis increases in low frequency fluctuations are correlated with increases in motor activity (replay-closed) despite the fact that on average motor activity was higher during closed-loop behavior and low frequency fluctuations were reduced. **B**(bottom): Furthermore changes in mean motor activity (replay-closed) and changes in pairwise correlations (replay-closed) (averaged over all pairwise interactions in a given trial) are not significantly correlated (r=0.03, p>0.5, Spearman’s rank correlation). (**C**) Filter describing behavioral feedback**.** A linear filter that describes *behavioral feedback* (E→E’) is also strongly negative. Following the kernel method outlined in the methods section we calculated the behavioral feedback as a direct filter between the closed loop environment and environment in the replay condition (E→E’). This also indicates that the closed-loop sensory feedback is negative. Note: the magnitude of this closed-loop sensory feedback estimated from the behavior is much greater than the cell self-feedback, reflecting the fact that cellular variability was much greater than variability across animals.
